# Phylogenomic analysis of a global collection of *Escherichia coli* ST38: evidence of interspecies and environmental transmission?

**DOI:** 10.1128/msystems.01236-22

**Published:** 2023-09-07

**Authors:** Piklu Roy Chowdhury, Priyanka Hastak, Matthew DeMaere, Ethan Wyrsch, Dmitriy Li, Paarthiphan Elankumaran, Monika Dolejska, Glenn F. Browning, Mark S. Marenda, Thomas Gottlieb, Elaine Cheong, John Merlino, Garry S. A. Myers, Steven P. Djordjevic

**Affiliations:** 1 Australian Institute for Microbiology & Infection, University of Technology Sydney, Ultimo, Sydney, New South Wales, Australia; 2 Central European Institute of Technology (CEITEC), University of Veterinary and Pharmaceutical Sciences, Brno, Czech Republic; 3 Department of Biology and Wildlife Disease, Faculty of Veterinary Hygiene and Ecology, University of Veterinary and Pharmaceutical Sciences, Brno, Czech Republic; 4 Biomedical Center, Charles University, Brno, Czech Republic; 5 Department of Clinical Microbiology and Immunology, Institute of Laboratory Medicine, The University Hospital, Brno, Czech Republic; 6 Asia-Pacific Centre for Animal Health, Melbourne Veterinary School, University of Melbourne, Parkville, Melbourne, Victoria, Australia; 7 Department of Microbiology and Infectious Diseases, Concord Hospital and NSW Health Pathology, Hospital Road, Concord, New South Wales, Australia; 8 Faculty of Medicine, University of Sydney, Sydney, New South Wales, Australia; University of Delhi, Delhi, India

**Keywords:** enteroaggregative *E. coli*, EAEC, ST38, One Health, *bla*_CTX-M_, genomic surveillance, phylogenomics, drug resistance, β-lactamase genes

## Abstract

We performed a comprehensive phylogenomic analysis of 925 extraintestinal pathogenic *Escherichia coli* (ExPEC) ST38 genomes from 38 countries and diverse hosts and sources. The phylogeny resolved two broad clades: A (593 strains; 91% human) and B (332 isolates; 42% human), each with distinct ST38 clusters linked to the carriage of specific *bla*_CTX-M_ alleles, often in association with other antibiotic resistance genes, class 1 integrons and specific plasmid replicon types. Co-carriage of *fyuA* and *irp2* virulence genes, a reliable proxy for carriage of the Yersinia high-pathogenicity island, featured in 580 (62.7%) genomes. ST38 lineages carrying combinations of ExPEC and intestinal pathogenic *Escherichia coli* virulence factors were also identified. The F plasmid replicon was identified in 536 (58%) genomes, and 112 of these (21%) carry *cjrABC-senB*, a virulence operon frequently identified in pandemic ExPEC sequence types. Most (108; 96.4%) *cjrABC-senB*+ ST38 isolates were from human and other sources, except food animals, and were associated with F5:A-:B10 (41 isolates), F1:A2:B20 (20 isolates), and F24:A-:B1 (15 isolates) F replicon types. ST38 genomes that were inferred to carry a ColV-F virulence plasmid (69; 7.4%) were mostly from human (12; 17.4%), avian (26; 37.7%), or poultry (10; 6.9%) sources. We identified multiple examples of putative inter-host and host-environment transmission events, where genomes differed by <35 SNPs. This work emphasizes the importance of adopting a One Health approach for phylogenomic studies that seek to improve understanding of antimicrobial resistance and pathogen evolution.

**IMPORTANCE:**

Extraintestinal pathogenic *Escherichia coli* (ExPEC) sequence type (ST) 38 is one of the top 10 human pandemic lineages. Although a major cause of urinary tract and blood stream infections, ST38 has been poorly characterized from a global phylogenomic perspective. A comprehensive genome-scale analysis of 925 ST38 isolate genomes identified two broad ancestral clades and linkage of discrete ST38 clusters with specific *bla*_CTX-M_ variants. In addition, the clades and clusters carry important virulence genes, with diverse but poorly characterized plasmids. Numerous putative interhost and environment transmission events were identified here by the presence of ST38 clones (defined as isolates with ≤35 SNPs) within humans, companion animals, food sources, urban birds, wildlife, and the environment. A small cluster of international ST38 clones from diverse sources, likely representing progenitors of a hospital outbreak that occurred in Brisbane, Australia, in 2017, was also identified. Our study emphasizes the importance of characterizing isolate genomes derived from nonhuman sources and geographical locations, without any selection bias.

## INTRODUCTION

Extraintestinal pathogenic *Escherichia coli* (ExPEC) infections are on the rise globally, remain a challenge to define genetically, and increasingly encode resistance to multiple, clinically important antimicrobials (1). The rapid increase in the frequency of drug-resistant ExPEC infections (2) has led to a bias in reporting *E. coli* displaying resistance to extended-spectrum β-lactams, carbapenems, and fluoroquinolones (1, 3), adversely influencing the understanding of ExPEC pathogenicity (3). For example, ExPEC ST73 and ST131 are the leading causes of ExPEC-associated urinary tract infection (UTIs) and bloodstream infections (BSIs) globally (4, 5), yet ST73 appears in only 99 PubMed entries compared to 1,289 entries for ST131 (16 March 2023). ExPEC strains are also the leading cause of urinary tract and bloodstream infections, meningitis, and ventilator-associated pneumonia in humans (6, 7).

Whole-genome sequencing (WGS) has enabled a deeper understanding of *E. coli* phylogeny (8) and an improved appreciation of hybrid *E. coli* lineages that do not fit pathotype designations (9). Only a handful of over 13,000 *E. coli* sequence types (STs) represent the pandemic lineages that are responsible for most extraintestinal *E. coli* infections that impact human health. These include ST131 (serotype O25b:H4), ST95 (O1/O2/O18:K1:H7), ST73 (O6:K2:H1), ST69 (O11/O17/O77:K52:H18), ST648 (O25a:H4), ST393 (O15:K52:H1), ST405 (various serotypes), ST117 (various serotypes), and ST10 (various serotypes) (1, 10, 11) while ST1193 (12, 13), ST8196 (14), ST410 (15), and ST457 (16) are considered emerging pathogens. Although *E. coli* ST410, ST167, and ST38 are frequently identified in epidemiological investigations of ExPEC infections, their reservoirs, host diversity, modes of dissemination, and plasmid carriage remain largely unexplored (11, 17). These are important observations because the carriage of F plasmid lineages can have a profound impact on ExPEC host range (18, 19). ST38 and ST410 have been primarily linked with the global dispersion of *bla*_OXA-48_-like genes (*bla*_OXA-48_, *bla*_OXA-181_, *bla*_OXA-232_, and *bla*_OXA-204_), one of the most rapidly dispersing carbapenemase gene families worldwide (3). ST38 is significant in this regard because of the carriage of a chromosomal copy of Tn*6237,* which has been reported from isolates in the United Kingdom (20), Columbia (21), the Czech Republic (22), and Lebanon (23). A chromosomal copy of Tn*6237* has also been found in *E. coli* ST69 in Egypt (24). It is notable that most ST38 strains that carry *bla*_OXA_-like genes often also carry extended spectrum β-lactamase (ESBL) genes.

Genomes of *E. coli* ST38 isolates retrieved from extra-intestinal and bloodstream infections frequently contain multiple antibiotic-resistance genes that either inactivate or reduce susceptibility to penicillins, aminoglycosides, trimethoprim, and cephalosporins (14, 25, 26). The lineage has also been associated with community-onset infections in the United States (27), Germany (28, 29), France (30, 31), Japan (32), Australia (14), Thailand (33), Saudi Arabia (34), and Pakistan (35). *E. coli* ST38 carrying genes encoding extended-spectrum β-lactamases has been isolated from retail raw chicken in Japan (36) and the United States (37). Although *E. coli* ST38 appears to have a relatively strong association with poultry (28, 38 – 40), they have also been isolated from cattle in Israel (41), retail meats (26), companion animals (42 – 44), the Australian silver gull (*Chroicocephalus novaehollandiae*) (45, 46), and from different environmental sources (47).

*E. coli* ST38 continues to rise as a significant cause of extraintestinal disease and has been linked to outbreaks. It survives in diverse hosts and environments, suggesting it is a generalist that can maintain or otherwise retain genes encoding resistance to clinically important antibiotics (CIA). Here, we performed genomic and phylogenetic analyses of 925 genomes of ST38 isolates from diverse geographic locations and sources, representing the largest analysis of ST38 to date. The data were used to investigate clade structure in relation to plasmid, serotype, antibiotic resistance, and virulence-associated gene (VAG) carriage. We also investigated evidence of clonal dispersal of isolates between humans, animals, and disparate environmental ecosystems.

## MATERIALS AND METHODS

### *E. coli* strains

A total of 23 *E. coli* isolates were sequenced in-house (seven from humans, nine from birds, and seven from dogs). Strains EC36_ST38, EC231_ST38, and EC274_ST38 (Table 1) were isolated from patients with bacteraemia at Concord Hospital in Sydney. HOS16, HOS46, HOS59, and HOS72 were obtained from patients at the Orange Base Hospital in regional New South Wales (NSW). The genome sequences of strains with EC and HOS prefixes were published recently (14, 48). The MVC isolates (Table 1) were part of a larger whole-genome sequencing study of 299 ExPEC isolates from the University of Melbourne, School of Veterinary Medicine. Isolates with a CE prefix (Table 1) were from a collection of *Enterobacteriaceae* recovered from cloacal swabs of silver gull chicks inhabiting three nesting colonies on the south-eastern coast of NSW, Australia (16, 49). Strains were routinely sub-cultured on Lysogeny Broth (LB) agar plates and incubated for 18 h at 37°C. For genomic DNA extraction, overnight cultures were grown in LB on a rotary shaker set at 2,500 rpm.

**TABLE 1:** Genotype of 23 Australian ST38 genomes sequenced in house

Strains	Source	Year of isolation	Serotype	Resistance genotype	Class1 integron and associated genes	Virulence genotype	Plasmid replicon	Virulence factors usually linked with IncF plasmids
EC231	Human	2014	O86:H18	*aadA5, aph3, dfrA17, mphA, strA, sul1, sul2, tetA, blaCTX-M-27*	*intI1△, IS26, qacEdelta1, sul1*	*eitA, eitB, eitC, eitD, fecA, fyuA, irp2, kpsMT(II), traT, yeeT*	IncFIB, IncFII	*iss*
EC274	Human	2015	O1:H15	*aac3, blaTEM-1, catA1, tetB*	*IS26*	*fimH, fyuA, ipaH, irp2,kpsM, kpsMT(II), papB, traT, yeeT*	Col(BS512), IncFIA, IncFIB, IncFII	*iss, iucB, iucC, iucD, iutA*
EC36	Human	2013	O1:H15	*aac3, aadA5, aph3, blaTEM-1, dfrA17, mphA, strA, sul1, sul2, tetA, blaCTX-M-15*	*intI1△, IS26, qacEdelta1, sul1*	*fimH, fyuA, ipaH,irp2, kpsMT(II), papB, yeeT*	Col(BS512), Col(MP18), IncFIB, IncFII, IncI1(Gamma)	*iss, iucB, iucC, iucD, iutA*
HOS16	Human	2007	O50/O2:H30	*aac3, aadA5, aph3, blaTEM-1, dfrA17, mphA, strA, sul1, sul2, blaCTX-M-14*	*intI1, qacEdelta1, sul1*	*eitA, eitB, eitC, eitD, fimH, fyuA, hek, ipaH, irp2, kpsMT(II), traT, yeeT*	IncFII, pO111_1	*iss*
HOS46	Human	2006	O50/O2:H30	*aac3, aadA5, aph3, blaTEM-1, dfrA17, mphA, strA, sul1, sul2, blaCTX-M-14*	*intI1, qacEdelta1, sul1*	*eitA, eitB, eitC, eitD, fimH, fyuA, hek, ipaH, irp2, kpsMT(II), traT, yeeT*	IncFII, pO111_1	*iss, sitA, sitB, sitC, sitD*
HOS59	Human	2007	O1:H15	*aac3, aadA5, aph3, blaTEM-1, dfrA17, mphA, strA, sul1, sul2, tetA, blaCTX-M-14*	*intI1△, IS26, qacEdelta1, sul1*	*fimH, fyuA, ipaH, irp2,papB, papF, traT, yeeT*	IncB/O/K, IncFIA, IncFIB, IncFII	*iss, iucB, iucC, iucD*
HOS72	Human	2007	O50/O2:H30	*aac3, aadA5, aph3, blaTEM-1, dfrA17, mphA, strA, sul1, sul2, blaCTX-M-14*	*intI1, qacEdelta1, sul1*	*eitA, eitB, eitC, eitD, fimH, fyuA, hek, ipaH, irp2, kpsMT(II), traT, yeeT*	IncFII, pO111_1	*iss, sitA, sitB, sitC, sitD*
CE1708	Bird	2012	O7:H18	*qnrS, blaCTX-M-15*	*–*	*fecA, fimH, ipaH, yeeT*	IncY	*iss, ompT*
CE1751	Bird	2012	O86:H18	*aadA5, blaTEM-1, catA1, dfrA17, mphA, sul1, tetB, tetD, blaCTX-M-14*	*intI1, IS26, qacEdelta1, sul1*	*fimH, fyuA, hek, ipaH, irp2, merA, traT, yeeT*	Col(MG828), IncB/O/K, IncK, IncFIA, IncFIB, IncFII	*iss*
CE1760	Bird	2012	O86:H18	*aadA2, aadA3, aadA8, aph3, blaSHV-1, blaTEM-1, catA1, dfrA12, mphA, strA, sul1, sul2, tetD*	*intI1, qacEdelta1, sul1*	*eitA, eitB, eitC, eitD, fimH, fyuA, hek, ipaH, irp2, kpsMT(II), traT, yeeT*	Col156, IncFIB, IncFII	*iss, sitA, sitB, sitC, sitD*
CE1860	Bird	2012	O7:H15	*blaBIL, blaCMY, blaLAT_1*	*–*	*fimH, kpsM, yeeT*	IncFIB, IncI1(Gamma)	*col1a-operon, iss, sitA, sitB, sitC, sitD*
1603H	Bird	2012	O86:H18	*aph3, blaTEM-1, catA1, dfrA7, strA, sul2, tetD*	*intI1*	*eitA, eitB, eitC, eitD, fimH, fyuA, hek, ipaH, irp2, kpsMT(II), papB, traT, yeeT*	Col156, IncFIB, IncFII	*iss, iucB, iucC, iucD, iutA, sitA, sitB, sitC, sitD*
1708H	Bird	2012	O7:H18	*qnrS, blaCTX-M-15*	*–*	*fecA, fimH, ipaH, yeeT*	IncY	*iss, ompT*
CE1577	Bird	2012	O102:H6	*aac3, aadA5, aph3, blaBIL, blaCMY, blaLAT_1, blaTEM-1, dfrA17, mphA, strA, sul1, sul2, blaCTX-M-14*	*intI1, IS26, qacEdelta1, sul1*	*eitA, eitB, eitC, eitD, fimH, fyuA, hek, ipaH, irp2, traT, yeeT*	IncFIB, IncFII, IncFII	*iroB, iroC, iroD, iroE, iroNsitA, sitB, sitC, sitD*
CE1871	Bird	2012	O102:H6	*aac3, aadA5, aph3, blaTEM-1, catA1, dfrA17, mphA, strA, sul1, sul2, tetD, blaCTX-M-14*	*intI1, IS26, qacEdelta1, sul1*	*fimH, fyuA, hek, ipaH, irp2, yeeT*	NP	*iss, sitA, sitB, sitC, sitD*
CE1882	Bird	2012	O50/O2:H30	*aac3, aadA5, aph3, blaTEM-1, dfrA17, mphA, strA, sul1, sul2, blaCTX-M-14*	*intI1, IS26, qacEdelta1, sul1*	*fimH, fyuA, hek, ipaH, irp2, kpsMT(II), yeeT*	NP	*sitA, sitB, sitC, sitD*
MVC219	Dog	2011	O7:H15	*aph3, blaTEM-1, strA, sul2*	*–*	*fimH, kpsM, kpsMT(II), yeeT*	Col(MG828), IncB, IncK, IncFIB, IncI2(Delta), IncX4	*sitA, sitB, sitC, sitD*
MVC224	Dog	2011	O1:H15	NP	*–*	*fimH, fyuA, ipaH, irp2, kpsM,*	NP	*iss, sitA, sitB, sitC, sitD*
MVC279	Dog	2012	O1:H18	*aph3, blaTEM-1, dfrA14, mphA, strA, sul2*	*intI1*	*fimH, fyuA, hek, ipaH, irp2, papB, yeeT*	NP	*iss*
MVC4	Dog	2008	O86:H18	*aac3, aadA2, aadA3, aadA8, aph3, blaTEM-1, catA1, dfrA12, mphA, strA, sul1, sul2, tetD, blaCTX-M-14*	*intI1, qacEdelta1, sul1*	*fimH, fyuA, hek, ipaH, irp2, yeeT*	NP	*iroC, iroD, iroE, iroN, iss*
MVC815	Dog	2008	O86:H18	*aac3, aadA2, aadA3, aadA8, aph3, blaTEM-1, catA1, dfrA12, mphA, strA, sul1, sul2, tetD, blaCTX-M-14*	*intI1, qacEdelta1, sul1*	*fimH, fyuA, hek, ipaH, irp2, yeeT*	NP	*iroC, iroD, iroE, iroN, iss*
MVC82	Dog	2009	O86:H18	*blaCTX-M-14*	*IS26*	*fimH, fyuA, hek, ipaH, irp2, yeeT*	NP	*iroC, iroD, iroE, iroN*
MVC99	Dog	2009	H15	*blaTEM-1, dfrA5, sul1, tetA*	*intI1, IS26, qacEdelta1, sul1*	*estA, fimH, ipaH, ompT, traT, yeeT*	IncFIB, IncFIC, IncY	*estA, estB, estC, hlyF, iroB, iroC, iroD, iroE, iroN, iss, iucA, iucB, iucC, iucD, iutA, ompT, sitA, sitB, sitC, sitD*

### Whole-genome sequencing

Genomic DNA for short-read sequencing was extracted using the ISOLATE II Genomic DNA kit (Bioline, Australia), following the manufacturer’s protocol. DNA was quantified using a Qubit fluorimeter and the dsDNA HS Assay kit (Thermo Fisher Scientific, Australia). The NucleoSpin Tissue kit (Macherey-Nagel GmbH & Co.) was used to extract genomic DNA from ST38 strains with the CE prefix, 1603H and 1708H (16). Whole-genome sequencing libraries for the HiSeq run were prepared from 2 ng of gDNA template with the Illumina Nextera XT kit and sequenced on an Illumina HiSeq 2500 v4 sequencer (Illumina, San Diego, CA, USA) in rapid PE150 mode (14). Raw reads were assembled into draft genomes using Shovill v1.0.4 (https://github.com/tseemann/shovill). GenBank accession numbers and assembly statistics for the 23 draft genome sequences from Australia are presented in Table S1. Genome sizes ranged between 4,966,866 bp and 5,545,072 bp (median 5,207,191 bp). With the exception of CE1577, the number of scaffolds per genome ranged from 78 to 321 (median 147). Read depth ranged from 8.2× to 33.2× (median 24×). The genome of isolate CE1577 comprised 535 contigs, and the coverage was low (2.5×).

Plasmid DNA sequences in EC36_ST38 and EC274_ST38 were resolved using hybrid assembly of short read and Pac-Bio long read sequences generated using a Pac-Bio microbial multiplex-sequencing protocol. Sequencing was performed on a Pacific Biosciences Sequel platform available at the Ramaciotti Centre for Genomics (UNSW). Genome sequences were co-assembled using Unicycler (version 0.4.7) (https://github.com/rrwick/Unicycler) as described previously (50).

### Phylogenetic analyses

**Fig 1:**
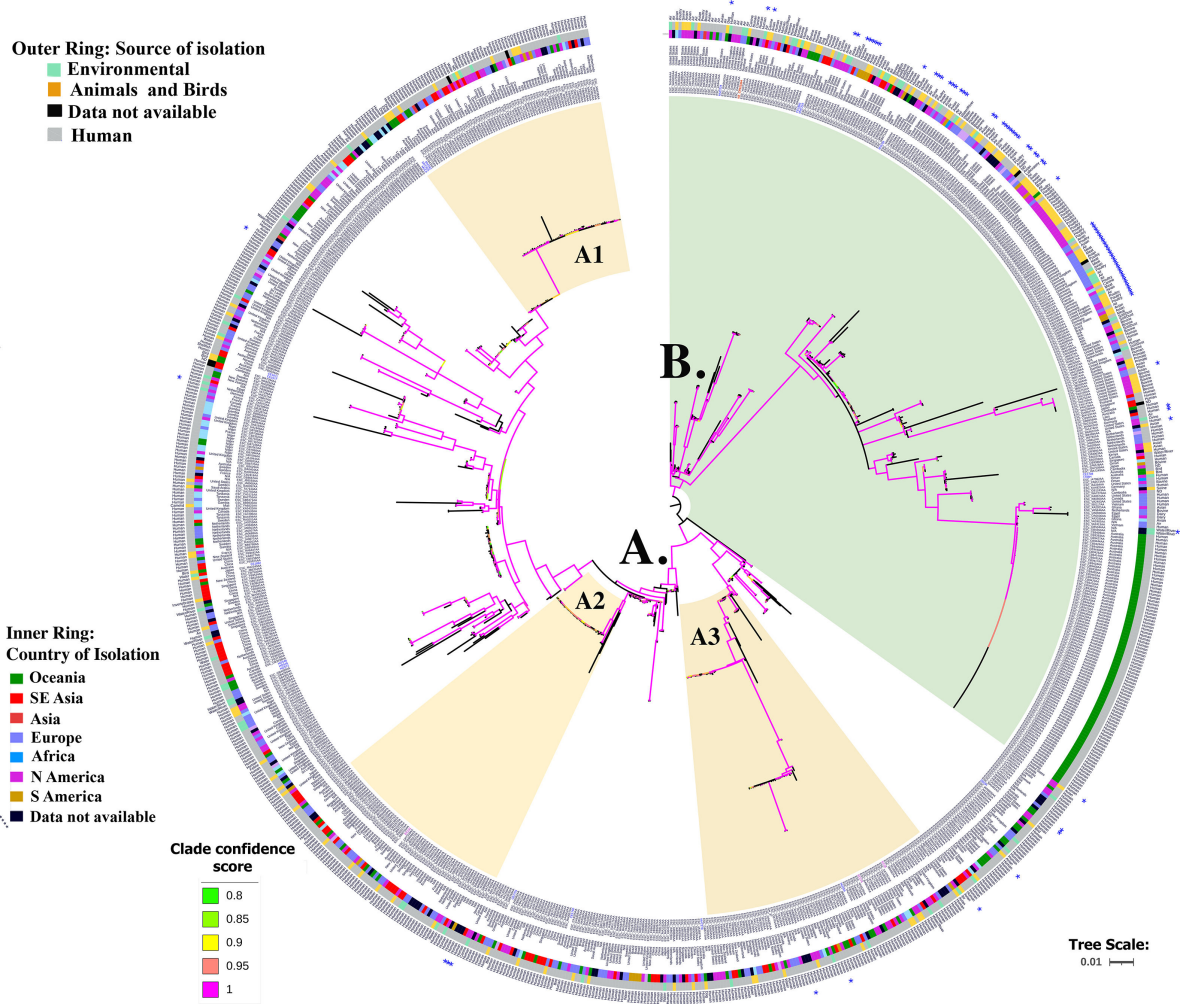
A midpoint-rooted phylogenetic tree representing the global phylogeny of 925 ST38 genomes. Clade confidence scores > 0.8 are indicated in different colors. Subclusters subjected to sub-phylogeny analyses are highlighted as colored strips. Metadata, presented as concentric circles (inside to outside), indicate (1) the country of isolation in text, (2) continent (indicated by different color codes along the inner circle), (3) human, animal or environmental niche indicated in the color-coded outer circle, (4) exact source of isolation as text, and (5) * indicates isolates that probably harbor a ColV-like plasmid.

Core genome alignment-based SNP phylogeny of the genomes was inferred with parSNP v1.2 (51) using the -c and -x flags to evoke forced alignment across collinear blocks and using recombination filtering to improve the accuracy of the estimated clonal ancestry. The closed and annotated genome sequence of *E. coli* ST38 strain 114 (NZ_CP023364) was recovered from the RefSeq database and was used as a reference for all pairwise SNP comparisons. Nine hundred and one ST38 genomes filtered for metadata defining source and/or country of isolation were downloaded from Enterobase on the 29 April 2020. The metadata associated with these genomes is presented as Table S2. Approximately 56% of the complete chromosomal sequence of the reference genome NZ_CP023364 (downloaded from RefSeq) was conserved in all genomes included in the analysis. Both variant and invariant sites within the conserved regions were aligned to deduce phylogenetic inferences. A total of 46,599 variant sites were utilized in the construction of the phylogenetic tree presented in Fig. 1. The matrix of variant sites per genome in the .vcf, file generated as a part of the parSNP, run was used to calculate pairwise SNP distances. To validate the overall topology of the phylogenetic tree and clustering of genomes clades and subclades, a second phylogenetic tree was constructed adopting a two-step procedure to generate a multi-sequence alignment (MSA) from the full genomic sequences of the 924 genomes using Snippy (v4.6.0) (52) against the single reference sequence (acc: NZ_CP023364.1). First, a simple Nextflow (v21.10.6) (53) script was used to perform a standard SNP analysis for each isolate against the reference using default options. Subsequently, a core MSA was generated using snippy core (default options). The full core MSA, which included gaps and invariant sites, contained 5,134,443 columns (9.93% gaps, 97.41% invariant sites) with 284,857 unique columns (patterns). From the full core MSA, a maximum likelihood phylogenetic tree (Fig. S1) was inferred using FastTree (v2.1.11) (54) using the model specification GTR + CAT and rescaled branch lengths (command line: “-gtr -gamma”). Following advice from the authors of FastTree for nearly identical sequences (https://darlinglab.org/blog/2015/03/23/not-so-fast-fasttree.html), a custom FastTree binary was compiled with the flags (“-DUSE_DOUBLE -O3 -finline-functions -funroll-loops -march = native”) under GCC (v8.5.0) (55).

Based on the clustering of genomes in both phylogenetic trees, three isolate clusters in clade A (named A1, A2, A3) and clade B were further selected (Table S3) for subphylogeny analysis using parSNP and using the same reference genome described above. The total number of variant sites used to infer the phylogeny is included in the supplementary figures (Fig. S2–S4). All phylogenetic tree figures were annotated in iTOL (56) and edited using Adobe Photoshop. parSNP was also used to infer phylogenetic relationships among plasmids. Linear alignment of closely related plasmids was drawn using BRIG (57).

For purposes of this study, the “genome clusters” (or monophyletic groups) identified for further analysis were defined as groups of more than 50 isolates from diverse collection sources, a confidence score or lowest common ancestral branch support value of 1, and similar resistance genotypes. Some variations in the content of resistance genes were tolerated, as the resistance genes are well known to be parts of accessory regions of the genome. Clones were defined as isolate pairs/clusters which had ≤35 pairwise SNP distance with the reference genome.

### Gene identification, pathogenicity index calculations, and MLST

The ResFinder, PlasmidFinder, and SeroTypeFinder databases were downloaded from the respective portals available through the Centre of Genomic Epidemiology website (http://www.genomicepidemiology.org/) and used to run BLASTn analysis on the HPC cluster at UTS. Outputs were filtered for >95% identity over >99% query length to identify positive hits. For *bla*_CTX-M_ and *bla*_OXA_ genotyping, stringency cut-off values were set to 100% identity over 100% query length. A pathogenicity index (PI) was calculated by dividing the total number of virulence genes present in the chromosome of an isolate by the total number of virulence genes (50) used in our in-house database of *E. coli* virulence gene sequences. ColV-like plasmids were identified following the criteria defined by Liu et al. in 2018 (58). For IncF replicon-based sequence type analysis (IncF RST), the database and software were downloaded and run locally on the UTS HPCC using default parameters. Preliminary genome annotations were generated using an online version of RAST (59). Putative antimicrobial resistance genes of interest were identified, curated manually, and subsequently confirmed by iterative BLASTn and BLASTp searches (60).

## RESULTS

### Geographical distribution and diversity between the sources of the strains included in the study

The 23 ST38 isolates from our in-house collection were sourced from seven humans, seven dogs, and nine Australian Silver gull (*Chroicocephalus novaehollandiae*) chicks (Table 1). We also included an analysis of 901 *E. coli* ST38 genomes (Table S2) downloaded from Enterobase, and a reference genome (NZ_CP023364.1) from GenBank for core-genome phylogeny analysis for a total of 925 genomes representing isolates from 38 countries:134 from Australia; 149 from the United States; 63 from China; 58 from Canada; 52 from the Netherlands; 43 from the United Kingdom; 37 from Sweden; 29 from France, 26 from Germany; 22 from Singapore; 21 from Brazil; 21 from New Zealand; 15 from Japan; 12 each from Vietnam, Niger, and Gambia; and 11 from Denmark. Of the remaining 185 isolates, a country of origin was not available for 106 genomes. Most (677 of 925; 73%) isolates were of human origin, followed by birds (67), poultry (31), air (41), cattle (22), water (25), dogs (15), gulls (9), soil/dust (8), and meat (8). Seven isolates had an unknown source. The remaining 15 isolates were from primates (3), swine (2), dairy (2), shellfish (2), a cat (1), a camelid (1), a sheep (1), a plant (1), an invertebrate (1), and a laboratory strain (1).

### Phylogeny of the global collection of ST38 genomes

Whole-genome alignment-based SNP phylogeny analyses using two different analytical pipelines were used to infer phylogenetic relationships between the 925 *E. coli* ST38 genomes (Fig. 1; Fig. S1) included in this study. The genomes clustered in two major clades, A and B. The overall topology of the trees generated using the two different SNP calling softwares (Fig. 1; Fig. S1) and the clustering of isolates into the two major clades were identical. The maximum pairwise SNP difference between isolates in the parSNP tree was 6,722 (Fig. 1). Clade A contained 593 isolates with a maximum of 4,501 pairwise SNPs with the reference genome, while clade B had 332 isolates with a maximum pairwise SNP divergence of 6,649. Clade A was not only dominated (91%, 538 of 593) by isolates from human infections but also included 21 environmental samples (air or water), 20 from birds and 7 from dogs. Despite the inclusion of 85 clonal isolates from a hospital outbreak in Queensland, Australian clade B was dominated (56.6%; 188/332) by isolates from non-human sources, (Fig. 1). Of the 188 non-human isolates in clade B, 56 were from birds, 31 from poultry, 38 from air, and 22 from cattle, with the rest from a wide range of sources including dogs, dairy, meat, soil, plant, and water (File S3). With the exception of the 2017 Australian epidemic clonal cohort (85 isolates) in clade B, clustering of genomes by geographic location was not evident.

### *In silico* serogroup profile analysis

A complete *in silico* O- and H-antigen profile could only be generated for 483 ST38 genomes (Table S3). Serotype O86:H18 was most prevalent (203 isolates) and was confined to clade A. Although most O86:H18 ST38 isolates were from human infections, six were from dogs, five were from birds, and three were from water. EC231, a community-onset blood stream infection (CO-BSI) isolate from our in-house collection, also had an O86:H18 serotype (14). O1:H15 (71 isolates) and O50/O2:H30 (56 isolates) were the second and third most prevalent serotypes, respectively. Of the 71 O1:H15 ST38 isolates, 62 clustered in clade A and all but four were from human infections. The nine O1:H15 isolates in clade B included five isolates from air, single isolates from a cat and a dog, and two from humans. Isolates with an O50/O2:H30 serotype were dispersed in smaller clusters within clade A, and all except six were from human infections. Three isolates collected from human urinary tract infections treated at a regional NSW hospital (HOS72, HOS46, and HOS16) and a gull chick (CE1882) in our in-house collection had an O50/O2:H30 serotype. The O-antigen type could not be determined for 103 isolates in clade B, all of which had an H9 flagellar antigen. This subset included the 2017 outbreak cluster (85 isolates) reported from a large hospital in Brisbane, Australia, and 18 other isolates from various countries and diverse non-human sources.

### Virulence gene profiling

All isolates were initially screened for the presence of 50 chromosomally located VAGs (Table S4) associated with ExPEC. Following the definition of PI defined for this study (see Materials and Methods), only 14 (0.15%) of the 925 *E. coli* ST38 genomes had a PI > 0.5 (Tables S3 and S4). Six of these 14 isolates with a PI >0.5 were obtained from human infections and clustered in clade A, while the remaining eight were in clade B and were obtained from birds or from air. All six human-associated isolates carried a *fimH*30 fimbrial adhesin gene variant. Isolates ESC_BB4897AA, ESC_RA5159AA, ESC_JB3298AA, and ESC_LA1335AA, which had the highest PI (~0.7), were isolated from birds, poultry, or air. A complete O- and H- antigen profile could not be generated for these 14 isolates, with the exception of ESC_JB3298AA, a poultry isolate from Spain, which had an O7:H18 serotype profile.

Consistent with most *E. coli* ST38 in the global collection, 17/23 Australian ST38 isolates harbored *fimH*, *fyuA*, *ipaH*, *irp2,* and *yeeT* genes (Table 1). Co-carriage of *fyuA* and *irp2* indicates that ST38 carries the Yersiniabactin HPI, a genomic island linked to virulence in ExPEC (61, 62). Isolate 1603H carried 13 of the 50 VAGs. Six isolates (four from humans, HOS16, HOS46, HOS72, and the CO-BSI isolate EC231), and two from silver gulls (CE1577 and CE1760) carried 10–12 VAGs (Table 1). Except for isolate CE1577, all *E. coli* ST38 isolates with 10 or more VAGs were serotype O86:H18 or O50/O2:H30. Table 1 also provides an account of genes that typically define accessory regions of a genome in the subset of 23 ST38 isolates included from our in-house collection. Significantly, O86:H18 and O50/O2:H30 isolates with more than 10 VAGs carry an IncF plasmid replicon. All isolates from human infections carried both *iss* (serum resistance) and *sit* (iron and/or manganese transport) genes except EC231 and HOS16, which had the *iss* gene only. EC274, EC36, HOS59, 1603H, and MV99 carried the *iuc* iron uptake operon, but only MV99, isolated from a dog in Australia, carries a ColV-F plasmid.

Some ST38 genomes are known to carry virulence determinants that define enteroaggregative *E. coli* (EAEC) including aggregative heat stable gene *astA;* the non-fimbrial adhesin gene *afaF*; iron transport genes (*shuA, shuT, shuX*); anti-aggregation protein gene *aap* (63); and the plasmid associated negative regulator, *aggR,* in the *aggR*-regulon (27, 64, 65). In our broader collection, *aggR* was identified in 99 isolates, all of which clustered in clade A and 93 were from human infections. An analysis (Table S3) undertaken to determine the presence of all seven EAEC-specific virulence genes revealed that *shuA and shuT* were present in 918 (99.24%) isolates, a result consistent with a recent study (63). In clade A, 11 isolates with *fim*H30 had five of the target EAEC genes (*app, aggR, astA, shuA, shuT*); 78 had four (*app, aggR, shuA, shuT*); and 10 carried *aggR*, *shuA,* and *shuT*. Given the presence of *aggR* in 99 isolates, we sought to determine the presence of pAA_Ec042-like EAEC plasmids (NC_017627.1) in the ST38 genomes but were unable to find a significant match.

Notably, clade A contained 117 isolates which carried *cjrAB*C-*senB* and either both or one of the Fe-transporter operon and *colE1* genes. These features are characteristic of pUT189-like virulence plasmids that dominate pandemic ExPEC lineages although the plasmid replicon types varied, and two isolates did not have a typeable plasmid indicating the possible location of the operon in the chromosome. A substantial proportion (34/120; 28.3%) of these isolates clustered in the A3 subclade (Fig. 1). Consistent with studies in other pandemic ExPEC lineages, ST38 isolates carrying F virulence plasmids with *senB* were from humans and other diverse sources but not from agricultural animal sources (13, 18).

### Plasmid replicon typing and prevalence of virulence plasmids

Plasmid replicon typing identified an IncF replicon in 58% (533/925) of the ST38 genomes (Table S3), often co-residing with a second or a third plasmid replicon. IncI replicons were identified in 218 genomes and col-like replicons in 221 ST38 genomes, respectively. The pMLST profiles of the ST38 genomes that carried a specific typeable F replicon in more than 10 isolates were F1:A-:B33 (57 isolates), F2:A-:B10 and F51:A-:B10 (41 isolates each), F24:A-:B1 (29 isolates), F1:A-:B23 and F30:A-:B- (28 isolates each), F1:A2:B20 (26 isolates), F4:A-:B1 (24 isolates), C4:A6:B26 (20 isolates), F64:A-:B1 (16 isolates), F2:A-B- (18 isolates), and C4:A-B1 (10 isolates). The remaining 195 isolates had 99 different F replicon types, with less than 10 representatives in each (Table S3). The *cjrAB*C-*senB* operon was present in 112 isolates with a typeable F replicon. The *cjrAB*C-*senB* operon was often associated with ST38 isolates that carried F51:A-:B10 (41 isolates), F1:A2:B20 (20 isolates), and F24:A-:B1 (15 isolates) replicons. Only 7 ST38 isolates carried a F29:A-:B10 replicon, typical of the pUT189 plasmid (66). Five of these (ESC_FB2091AA, ESC_GB8666AA, ESC_PA2128AA, ESC_TA1975AA, and ESC_GA8819AA; all sourced from humans) contained the entire complement of genes required for conjugative transfer of pUT189, likely indicating the presence of pUT189 or a close variant of it (66, 67).

Of the 78 ColV-F plasmids (8.4% of the collection) identified in the study (Table S3—genomes in bold fonts with *superscript), 22 carried an F4:B1 replicon, 9 have a C4:B1 replicon, and 4 had an F24:B1 replicon, with F34:B1, F64:B27, and F76:B1 replicons identified in three isolates each. Ten isolates could not be typed using the standard pMLST genes for F replicon typing, while the rest had variable IncF sequence types (Table S3—genomes in bold fonts with *superscript). Twenty of the 22 isolates with the F4:B1 replicon also harbored IncK and pO111_1-like plasmid replicons and the β-lactamase genes *bla*_BIL-1_ (encoding an extended-spectrum beta-lactamase), *bla*_LAT-1_ (cephalosporin resistance), and *bla*_CMY_ (Table S3). All 22 ST38 isolates with an F4:B1 replicon clustered in clade B and were from nonhuman sources and from different European countries.

### Complete sequence of plasmids assembled from multiple drug-resistant ST38 isolates EC36 and EC274 recovered from sydney hospital

#### F plasmids

F plasmid sequences from bacteraemia isolates EC36 and EC274 were resolved using a hybrid assembly as described previously (68). EC274 contained a 104,654 bp, F48:A1:B49 plasmid (pEC274F) that shared >99.9% sequence identity (along 91% query length) with pCRE1.1 (CP034396.1) and pCRE10.1 (CP034401.1). *cjrABC-senB* or other genes found on pUTI89 or ColV F virulence plasmids were not found on this plasmid. However, the strain carried *fimH*, *fyuA*, *irp*2, *ipaH*, *kpsM*, *kpsMT(II*), *papB*, *traT*, and *yeeT,* as well as *iss*, *iucBCD,* and *iutA*. The entire repertoire of resistance genes (*aac3, bla*_TEM-1_ *, catB1, tetB*) identified in EC274 and the class one integron resided on pEC274F.

In EC36, the F29:A-:B34 plasmid could not be completely assembled but resolved in two scaffolds of 75,006 and 30,182 bp (combined length of 105,118 bp) with all the resistance genes and the class 1 integron located on the 30 kbp scaffold. The plasmid carried the *cjrABC-senB* operon, as well as the *colE1* and iron-transporter-associated virulence genes found on the pUT189 plasmids, but the conjugation module was absent. It is notable that pUTI89-like plasmids that carry a complex resistance region typically house deletions in the plasmid *tra* genes (13). Pairwise alignment of the two IncF plasmids in EC36 and EC274 with genomic scaffolds of EC231 did not reveal significant homology.

#### I plasmids

**Fig 2:**
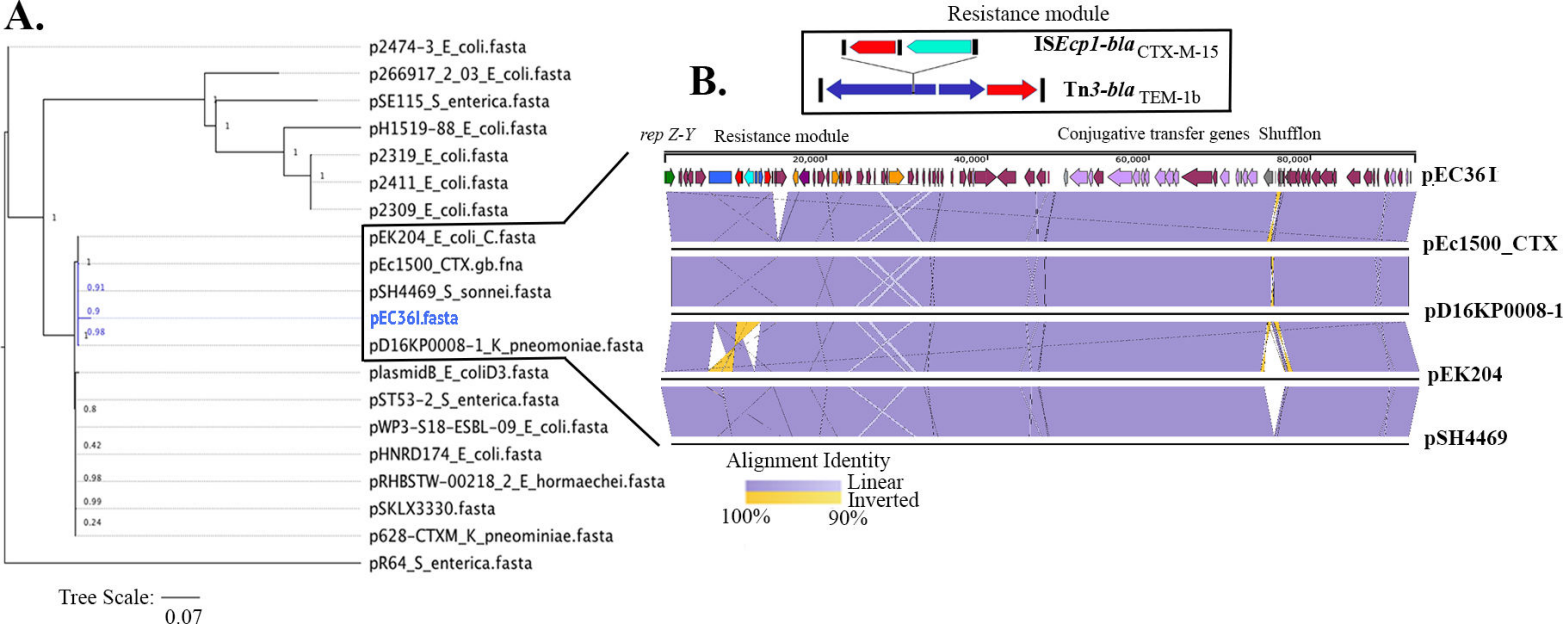
(**A**) Phylogenetic analysis of IncI plasmids. (**B**) Linear map of pEC36I with gene families coded in different colors. Red arrows indicate resistance genes, green arrow indicates the *rep A* gene, aqua arrows indicate mobile genetic elements, violet arrows indicate genes required for conjugative transfer, gray arrows indicate the shufflons, and purple arrows indicate backbone genes, including hypotheticals. The lines below represent the four most closely related IncI plasmids and their alignments with pEC36I. The alignment identities are presented as violet and yellow shades. The inset details the genetic context of the *bla*_CTX-M-15_ gene.

Isolate EC36 had an IncI1-ST16 plasmid (pEC36I) which was 93,000 bp in length and had >99.85% identity (over 92% query length) with plasmid pSKLX3330 (KJ866866.1) from *E. coli* strain SKLX3330 from China. The *bla*_CTX-M-15_ gene located on pEC36I was linked to an IS*Ecp1* module flanked by a 5 bp TATTG direct repeat (Fig. 2 inset). The pEC36I plasmid also hosts a Tn*3* transposon containing *bla*_TEM-1b_ (Fig. 2B). A BLASTn analysis of pEC36I identified 18 plasmid sequences with >98% sequence identity over >90% of the query length. The plasmids were linked to diverse bacterial genera and different geographical regions. Phylogenetic analysis of pEC36I revealed that it shared a most recent common ancestral relationship with pD16K0008-1 (*Klebsiella pneumoniae*), pSH444469 (*Shigella sonnei*), and pEc1500_CTXM and pEK204 from *E. coli* (Fig. 2A). pD16K0008-1 plasmid (GenBank accession CP052382.1) was from a *K. pneumoniae* blood stream infection in a Korean patient, while carriage of pSH4469 (GenBank accession KJ406378.1) was associated with a *S. sonnei* outbreak in Korea in 2008 (15). The plasmid backbone sequence of pSH4469 shared 99.97% identity with over 97% of the length of pEK204 (GenBank accession number EU935740.1), a plasmid that carries *bla*_CTX-M-3_ and was associated with an ST131 outbreak in the United Kingdom. Notably, pEc1500_CTXM (GenBank accession number CP040270.1), isolated from a marine bivalve mollusc in Norway, clustered in the same subclade (Fig. 2A). Sequence alignment of the four plasmids with pEC36I showed that they were almost identical with some variations in the shufflon (Fig. 2B).

The entire collection of 925 genomes was screened for the presence of pEC36I-like plasmids. Table S5 lists 173 genomes that harbored an IncI1 plasmid that shared >50% of the plasmid backbone with pEC36I, including 85 isolates from the *bla*_OXA-181_ outbreak cluster from Australia. Most of the isolates in the group resided in clade B and were sourced from human infections, but some isolates were from nonhuman sources. We also compared pEC36I with pMS1441A, the IncI1 plasmid from an Australian ST38 isolate that caused a hospital outbreak in Queensland (69). Although pMS1441A had >98.6% identity over 82% of the length of pEC36I, the IS*Ecp1-bla*_CTX-M-15_ module and the Tn*3* transposon, along with five other conjugation and hypothetical genes, were absent in pMS1441A.

#### Col plasmids

The ColBS512 plasmid found in EC36 (EC36_ColBS512) was 2,089 bp in length and 99.38% identical to plasmid pC (CP029795.1) from *S. sonnei* strain 4303. The ColBS501 plasmid in EC274 (EC274_ColBS512) was 2,101 bp in length and 99.9% identical to pColBS512 (CP058804.1) from *K. pneumoniae* strain BA27935 and p47733 (CP050362.1) from *K. pneumoniae* strain 47733. EC274 also carried a P1 phage-associated plasmid that carried toxin-antitoxin genes associated with phages as well as *phd*, *doc,* and conjugation transfer genes identified in F plasmids. The phage-associated plasmid has not been reported in *E. coli* ST38 previously.

### Genealogy and relative distribution of ESBL genes of isolates in clusters

The correlation of core-genome phylogeny with resistance genotypes (prevalent in accessory regions of the E. *coli* genomes and presented in Table S3) led us to further investigate three clusters of ST38 genomes within clade A, presented here as A1, A2, and A3 (Fig. 1; Fig. S1; Table S3). All three clusters had greater than 50 member isolates and were not restricted to human clinical sources only. Interestingly, these clusters were dominated by isolates with allelic variants of the CTX-M-type extended-spectrum β-lactamase gene, *bla*_CTX-M-14_ , *bla*_CTX-M-15_, and *bla*_CTX-M-27_, most frequently reported in relation to intractable outbreaks in human clinics but were not restricted to isolates sourced from humans only. The *bla*_CTX-M-14_ and OXA-181-associated outbreak clones reported recently from Brisbane, Australia, (69) were neatly clustered within clade B, which was dominated by isolates from nonhuman sources.

Cluster A1 had 70 isolates which assembled into a subclade (clade confidence score = 1) and pairwise SNP distance of ≤1,219 (Fig. 1; Fig. S1; Table S3). The most striking feature of this cluster was the presence of *bla*_CTX-M-14_ allele in all 70 isolates. To better resolve the phylogenetic distances, a sub-phylogeny analysis (Fig. S2) led to the identification of four groups of isolates with ≤25 SNPs in the core genome collected from diverse geographic areas and different hosts. Group I consisted of MVC4, MVC82, MVC815 (maximum pairwise SNP distance = 14) from dogs and three isolates (ESC_CB7213, ESC_PA4047, and ESC_RA0380) from humans with ≤20 SNP differences. Isolates in this cluster lacked a detectable plasmid replicon. Except for ESC_RA0380 (Sweden), all genomes were Australian. Group II consisted of three genomes, ESC_WA8254, ESC_FB9093, and ESC_MA2356, collected from different countries, but had ≤5 pairwise SNP differences in their core genomes. While the first two were sourced from human infections, isolate ESC_MA2356 was collected from water and had acquired a plasmid. Group III comprised six isolates from Brazil, two from dogs and four from humans, with ≤24 SNPs. Group IV comprised two isolates from human infections, one from Canada (ESC_GB3907) and one from France (ESC_CB2280), and a single bird isolate (ESC_TA2324) from Australia with ≤24 SNP differences.

Cluster A2 had 57 isolates, all except one contained the *bla*_CTX-M-27_ extended spectrum β-lactamase gene (Table S3) and pairwise SNP distance of ≤31. For the sub-phylogeny analysis of this cluster (Fig. S3), we additionally included 10 isolates, two of which had other allelic variants of the *bla*_CTX-M_ gene, while the rest did not have any CTX-M variant, but shared the closest common ancestral node (Fig. 1; Fig. S1). As evident in Fig. S3, the 57 isolates with *bla*_CTX-M-27_ in cluster A2 grouped with only 5 (of the 10 additional) isolates that did not have any *bla*_CTX-M_ variant and ESC_CB2284AA which had the *bla*_CTX-M-15_ gene. Here, we draw attention to three groups of isolates (Fig. S3). Group I consisted of a bird isolate from Australia (ESC_JB8396) and a human isolate from China (ESC_FB7958) with identical plasmid profiles, *bla*_CTXM-27_ and ≤20 SNP differences in the core genome. Group II comprised three human isolates from Canada (ESC_FB9365), Sweden (ESC_UA7319), and Australia (EC231) each with *bla*_CTXM-27_ that differed by six SNPs. All three isolates carried an IncF plasmid, and the Canadian isolate carried an additional ColB plasmid. The third cluster with *bla*_CTXM-27_ comprised just two isolates, 12 SNPs apart. One was a human clinical isolate (ESC_GB9439) from Singapore and the other was an isolate from water in the United States (ESC_RA6707).

We additionally conducted a sub-phylogeny analysis with a more divergent cluster, A3 (Table S3), which had 85 isolates and all variants of the *bla*_CTX-M_ genes, including *bla*_CTX-M3_ and *bla*_CTX-M55_, but predominantly sourced from human infections (Fig. S4) from geographically separated regions. All 85 isolates appeared to have clustered as a clade in Fig. 1; Fig. S1, with a confidence score of 1 but had ≤3,635 pairwise SNP differences. The sub-phylogeny analysis (Fig. S4) resolved the isolates into distinct subclades, with the majority of isolates having *bla*_CTXM-27_ clustering together, while isolates with the *bla*_CTXM-15_ and *bla*_CTXM-14_ variants appearing sporadically in different subclades. However, we were able to identify two isolate pairs and a group of three isolates which were clonal (following parameters defined in the methods section). Group I had two human clinical isolates, ESC_XA7257 (from Vietnam) and EC36 (from Sydney), which differed by two SNPs in their core genomes and had similar profiles for resistance and virulence genes (Table S3) and identical (F and I) plasmid replicon types. Interestingly, isolate EC36 had the *bla*_CTX-M-15_ gene while isolate ESC_XA7257 did not. However, we have demonstrated above that the *bla*_CTX-M-15_ gene in EC36 was an IS*Ecp1*-mediated insertion in the IncI plasmid with genetic signatures suggesting a local acquisition.

Group II comprised two human isolates carrying the *bla*_CTX-M-14_ allele that differed by 35 SNPs, one was from China (ESC_JB8356) and the other from regional NSW in Australia (HOS59). Each had identical resistance and virulence profiles (Table S3). Group III comprised three isolates, ESC_YA1551, ESC_MA2322, and ESC_IB9015, collected from a human clinical infection in Switzerland, a water sample from Japan and a human clinical sample from Australia, respectively. While the Japanese and the Swiss isolates had one SNP difference between them, the Australian isolates had five SNP differences. The isolates had identical plasmid profiles and had very similar resistance genotypes (Table S3), except the environmental isolate from Japan and the clinical isolate from Australia had the *bla*_CTX-M-27_ gene, while the clinical isolate from Switzerland did not have any *bla*_CTX-M_ allele.

The sub-phylogeny and genotype analysis of all 332 isolates in clade B (Fig. S5) revealed evidence of inter-species clonal transmission events. The paired isolate in group I, ESC_WA5522 (human) and MVC224 (dog) in Australia, differed by 14 SNPs and displayed similar resistance genotype profiles but different plasmid profiles. Group II comprised three Australian isolates, one from a seagull (CE1860) and two from humans (ESC_WA5556 and ESC_WA5549). The gull isolate was indistinguishable from ESC_WA5556 and differed from ESC_WA5549 by only four SNPs. The isolates had identical plasmid profiles but did not have a *bla*_CTX-M_ gene. Group III comprised nine ST38 isolates, including two from silver gulls [CE1708 (49) and 1708H] in Australia, one from river water in Canada (ESC_KP1033), and one from a cow in Japan (ESC_QA7509). This set of ST38 genomes from disparate sources and geographical regions had less than 20 SNP differences and displayed varied plasmid profiles. Group IV included a subset of five isolates (ESC_XA2030, ESC_GB4263, ESC_GB5344, ESC_VA2413, and ESC_VA2482), all except ESC_VA2413 from environmental sources. The maximum phylogenetic distance between isolates is 30 SNPs. These isolates had the H9 flagellar antigen, an IS*Ecp1*-linked *bla*_CTXM-15_, and all except ESC_VA2413 (sourced from human infection) had a low pathogenicity index. ESC_GB4263 and ESC_VA2413 (human, Vietnam) deserve special mention as their core genomes differed by less than 20 SNPs from a representative (ESC_CB9472) of the ST38-*bla*_OXA-181_ outbreak cluster (85 isolates) and had near identical genotype profiles, including chromosomally located genes and plasmid profiles. Based on our analysis, ESC_GB4263 and ESC_VA2413 may represent progenitors of a hospital outbreak strain in Brisbane, Australia, (CARAlert2017) in 2017.

## DISCUSSION

Here, we present the first large-scale phylogenomic analyses of 925 geographically dispersed *E. coli* ST38 isolates from multiple sources. Human isolates dominated the collection, but genomes from diverse non-human sources, including food (poultry bovine, swine) and companion animals (dog), the Australian silver gull (*Chroicocephalus novaehollandiae*), wildlife, meat, soil, air, plants, and water were included, providing a One Health perspective. We found numerous examples of ST38 isolates from different geographic locations and multiple sources that had less than 35 SNP differences in their core genome, some with near identical accessory gene content (plasmid and resistance gene cargo), indicating clonality and evidence of interhost strain sharing. Such associations would not be evident if the collection was restricted to isolates carrying ESBL or other genes conferring resistance to medically important antibiotics only. Furthermore, we provide evidence that near identical plasmids are likely shared between ST38 *E. coli*, *Shigella sonnei,* and *Klebsiella pneumoniae*.

ST38 displays substantial F plasmid replicon diversity. Nonetheless, 112 isolates carried F plasmids with the *cjrABC-senB* operon, and these displayed 23 different F replicon sequence types. Notably, pUTI89 and related F plasmids with replicon type F29:A-:B10 were poorly represented in ST38, but *cjrABC-sen*B^+^ F plasmids were associated with 112 ST38 isolates (18, 19). These were primarily sourced from humans, but five were from birds and one from water. Of 925 ST38 isolates, 68 (7.4%) carry ColV and related F virulence plasmids. These plasmids have diverse replicon sequence types with F4:A-:B1 identified in 22/68 (32.4%) isolates. These were from varied sources including 10 from poultry and 26 from different avian sources, details of which were not available in Enterobase.

Our analysis identified two major ST38 clades. Clade A with 593 isolates, was predominantly sourced from humans, while clade B comprised 332 isolates, of which 189 (57%) were from a range of non-human sources (Fig. 1). Included in our analyses were 157 ST38 isolates of Australian origin, of which 23 isolates were sequenced in-house. A significant proportion (85 of 157) of the Australian isolates was representatives of an ST38 outbreak (May to July, 2017) in Brisbane, Australia (69) (CARAlert 2017). The genomes of these 85 isolates were essentially indistinguishable (≤5 SNPs), with identical plasmid replicons (IncX3 and IncI1) and with genes (*bla*_OXA-181_ and *bla*_CTX-M-15_) encoding resistance to the same repertoire of CIA. The *bla*_OXA-181_ and *bla*_CTX-M-15_ genes in the outbreak cluster were located on an IncX3 plasmid, while the quinolone resistance gene, *qnrS1,* was on an IncI1 plasmid (69). An ST38 isolate from an index patient, who was hospitalized in Vietnam immediately prior to coming to Australia for further medical treatment in the month preceding the outbreak, carried a combination of these genes (69). For several months, the infection spread undetected, affecting more than 70 patients in multiple wards in a major tertiary hospital in Brisbane. WGS was used to recommend infection control measures (69). Our study identified a sub-clade of five isolates in clade B including two isolates, ESC_GB4263 from water/sewage (country unknown) and ESC_VA2413 from a patient in Vietnam, which appeared to be potential progenitors of the outbreak cluster. Our hypothesis is based on the observation that the genomes of these isolates differ by only 12 and 20 SNPs, respectively, from ESC_CB9472, a representative of the outbreak cluster. Our analysis is consistent with the study of Roberts et al. (69), which independently identified ESC_VA2413 as a progenitor but isolate ESC_GB4263 from water/sewage was not identified by Roberts et al. (69). We provide evidence that ESC_GB4263 and ESC_XA2030 (avian isolate from Ghana) in the sub-clade also carried an identical IncI plasmid replicon and *bla*_CTX-M-15_ gene profile indicating that the outbreak clone is likely dispersed globally. We also aligned the complete sequence of IncI plasmid pEC36I from EC36 (a bacteraemia isolate from Sydney hospital) with the genome sequences of ESC_GB4263 and ESC_VA2413 and ESC_CB9472AA from the Australian ST38-*bla*_OXA-181_ outbreak (data not presented). With the exception of Tn*3* associated TEM-1b module (Fig. 2B) in plasmid pEC36I, the entire plasmid sequence aligned to ESC_VA2413 contigs sourced from a human infection in Vietnam, while the environmental isolates ESC_GB4263 and ESC_CB9472 did not align to portions of the transfer region of pEC36I in addition to the Tn*3* associated TEM-1b module. These observations suggest that subtle differences can be found in IncI1-like plasmids in ST38 recovered from diverse sources.

*E. coli* ST38 is one of a number of *E. coli* STs recognized as leading causes of extraintestinal disease (3). Drug-resistant variants of ST38 seem to be reported frequently from Asia, the Middle East, and less often from North America (3). As of 24 December 2020, there were only 2,141 ST38 genomes in Enterobase, representing only 1.3% of the 159,562 *E. coli* genomes deposited in the database. Studies reporting the most frequent *E. coli* sequence types from consecutively collected, non-duplicate clinical samples, unbiased by preselection based on drug resistance, is rarely identified in the ST38 literature (70). A recent US-based, nationwide surveillance study found that clonal complex (CC) ST38 accounted for 4.8% of *E. coli* causing UTIs and 6.2% of those causing BSIs (71). Another recent study of ceftriaxone-resistant *E. coli* isolated predominantly from urine samples and spanning 2013–2018 in western New York, identified ST38 as the second most prevalent sequence type (15.7%) after ST131 (46%) (27). While *bla*_CTX-M-15_ was the main gene held responsible for encoding resistance to ceftriaxone in the western New York study, carriage of *bla*_CTX-M-27_ was also frequently identified in association with the carriage of distinct plasmids. In Australia, however, the repertoire of resistance genes that confer resistance to clinically important antibiotic genes appears to be diverse (Table S3). The bacteraemia isolates EC36 and EC231 from patients at the Concord Repatriation General Hospital in Sydney carried *bla_C_*_TX-M-15_ (EC36) and *bla_C_*_TX-M-27_ (EC231) (14) and the clonal ST38 outbreak from Brisbane carried *bla*_CTX-M-15_ and *bla*_OXA-181_ (69).

ST38 clades A and B each include multiple subclades that carry different allelic variants of *bla*_CTX-M_. For example, isolates in cluster A1 predominantly carry *bla*_CTX-M-14_, while isolates in cluster A2 predominantly carried *bla*_CTX-M-27_, irrespective of the country and source of isolation. A recent study suggested F2:B10 plasmids are the major disseminator of *bla*_CTX-M-27_ in ST38 in the United States (72). We identified 40 genealogically linked ST38 genomes that carry F plasmids with an F2:A-:B10 replicon type and *bla*_CTX-M-27_ from disparate geographical regions. These 40 isolates clustered in cluster A2 raising the possibility that F2:B10 plasmids and close variants are disseminated globally (72). The relative advantages of carrying different *bla*_CTX-M_ are not well known, but it has been posited that carriage of *bla*_CTX-M-27_ may confer additional activity against ceftazidime (73). Human clinical ST38 isolates are notable for their capacity to carry genes encoding resistance to clinically important antibiotics, including different allelic variants of the *bla*_CTX-M_ gene (31, 32, 74 – 79). Our data additionally show that the IncI1 plasmid pEC36I and its close variants are distributed across multiple Australian states and globally within ST38 genomes (Table S5).

ST38 carries fewer ExPEC-associated virulence genes than ST131 although the two have similar *in vitro* adhesion, invasion, and serum resistance phenotypes (80). ExPEC derived from fecal *E. coli* populations and intestinal pathogenic *Escherichia coli* (IPEC) that carry ExPEC VAGs are being reported more frequently (81, 82). Several studies have described ST38 isolates that carry virulence genes indicative of enteroaggregative *E. coli* (EAEC), an established IPEC pathotype (27, 65). Of 11 *aggR* ^+^ *E. coli* identified in a screen of 359 ESBL-positive *E. coli* isolates, six were ST38, and all were from extraintestinal sites (65). The *aagR* gene is considered a key EAEC VAG, but the definition of EAEC at the molecular level remains a challenge (83). Chattaway et al. (65) suggested that ST38 has acquired diverse VAGS, including the EAEC plasmid, which enhances their ability to colonize the gut, and concluded that EAEC ST38 may represent ExPEC associated with urinary tract infections. Others have also demonstrated carriage of EAEC VAGs by ST38 isolates (27, 81, 83). It has been suggested that ExPEC VAGs may enhance the capacity of EAEC to cause UTI (64). ST38 isolates recently characterized in New York did not carry the *aggR* or *aaf* EAEC VAGs, but they did harbor *afaF-III*, from the Afa/Dr adhesin family. *E. coli* expressing Afa/Dr adhesins is known to be clinically relevant in the context of UTIs (84). Finally, ST38 typically carries the Yersinia High-Pathogenicity Island, a noted ExPEC virulence island (61, 62). Our analysis revealed that ST38 had an unremarkable pathogenicity index when chromosomally located *E. coli* virulence genes were considered. However, we also demonstrate that ST38 *E. coli* carries diverse IncF, IncI, and IncX3 plasmids, and virulence genes associated with some specific pMLST-types, indicating a gradual influx of plasmid-associated virulence genes in these genomes. While ST38 does not appear to be a major host for pUTI89-like virulence plasmids that dominate various ExPEC sequence types (60), carriage of *cjrABC-senB* virulence genes on F plasmids was not uncommon in the collection of ST38 analyzed in our study. Our data additionally reveal that ST38 has readily disseminated within and between continents and between different hosts in the environment. Therefore, ST38 should be monitored beyond virulence-associated genes that define the *E. coli* pathotypes, including plasmid-associated virulence markers.

The major strength of this study is the identification of clonal pairs/isolate groups defined as isolates with <35 pairwise SNPs that reside within diverse clusters in the two major clades. Our data provide evidence of interhost as well as environment to animal/human transmission. Our observation is supported by a combination of data acquired from SNP-based core genome phylogeny and accessory genome content analysis. We acknowledge that the genomes included in this analysis were from a publicly available database (Enterobase) and with that comes inherent sampling bias that favors isolation from humans with illness, as well as isolates bearing clinically important resistance genes, and isolations reported from developed countries. Despite this limitation, our sample cohort included as much source and geographic diversity in sample size as possible, which helped us decipher important overall trends. Two different analytical approaches were used to interrogate phylogenetic inferences, conferring robustness to our analyses. In conclusion, ST38 is an unusual yet important ExPEC sequence type that demonstrates a propensity to carry ExPEC and IPEC VAGs, often aided by virulence-associated plasmid acquisition. *E. coli* ST38 not only causes a significant disease burden to humans and animals but is also readily detectable in non-clinical sources including animals (wild and domesticated), aquatic environments, and in wastewater streams. Our study highlights the importance of adopting genomic surveillance-based One Health approaches in understanding and predicting the evolution of antibiotic resistance and virulence gene acquisition and for interhost and other transmission pathways. Such One Health perspectives are critical to impede the rapid evolution of intractable variants of pathogenic bacteria with downstream ramifications in global healthcare practices.

## Data Availability

The assembled long-read genome sequences of EC36_ST38 and EC274_ST38 were submitted to GenBank under accession numbers JAGDML000000000 and JAGDMM000000000, respectively.

## SUPPLEMENTAL MATERIAL

Figure S1 msystems.01236-22-s0001.pdf Midpoint rooted maximum-likelihood phylogenetic tree generated using SNIPPY as the SNP caller which supports the overall topology of the tree presented in Figure 1.

Figure S2 msystems.01236-22-s0002.pdf Midpoint rooted maximum-likelihood phylogenetic tree representing geneology of 70 isolates in cluster A1.

Figure S3 msystems.01236-22-s0003.pdf Midpoint rooted maximum-likelihood phylogenetic tree representing geneology of 67 isolates in cluster A2.

Figure S4 msystems.01236-22-s0004.pdf Midpoint rooted maximum-likelihood phylogenetic tree representing geneology of 85 isolates in cluster A3.

Figure S5 msystems.01236-22-s0005.pdf Midpoint rooted maximum-likelihood phylogenetic tree representing geneology of 332 isolates in clade B.

Table S1 msystems.01236-22-s0006.xlsx Assembly statistics of genomes sequenced in-house.

Table S2 msystems.01236-22-s0007.xlsx Metadata of genomes downloaded from Enterobase.

Table S3 msystems.01236-22-s0008.xlsx Genotype summary of genomes included in this study.

Table S4 msystems.01236-22-s0009.xlsx Virulence profile of genomes included in the study.

Table S5 msystems.01236-22-s0010.xlsx Genomes with IncI-like plasmid backbone.

ASM does not own the copyrights to Supplemental Material that may be linked to, or accessed through, an article. The authors have granted ASM a non-exclusive, world-wide license to publish the Supplemental Material files. Please contact the corresponding author directly for reuse.
