# Supplementary material for: Phylogenomic analysis of a global collection of Escherichia coli ST38: evidence of interspecies and environmental transmission?
Source: mSystems. 2023 Sep 7;8(5):e01236-22. doi: 10.1128/msystems.01236-22 (PMC10654095; doi:10.1128/msystems.01236-22)
Supplement: Figure S1 — Midpoint rooted maximum-likelihood phylogenetic tree generated using SNIPPY as the SNP caller which supports the overall topology of the tree presented in Figure 1. [file msystems.01236-22-s0001.pdf]

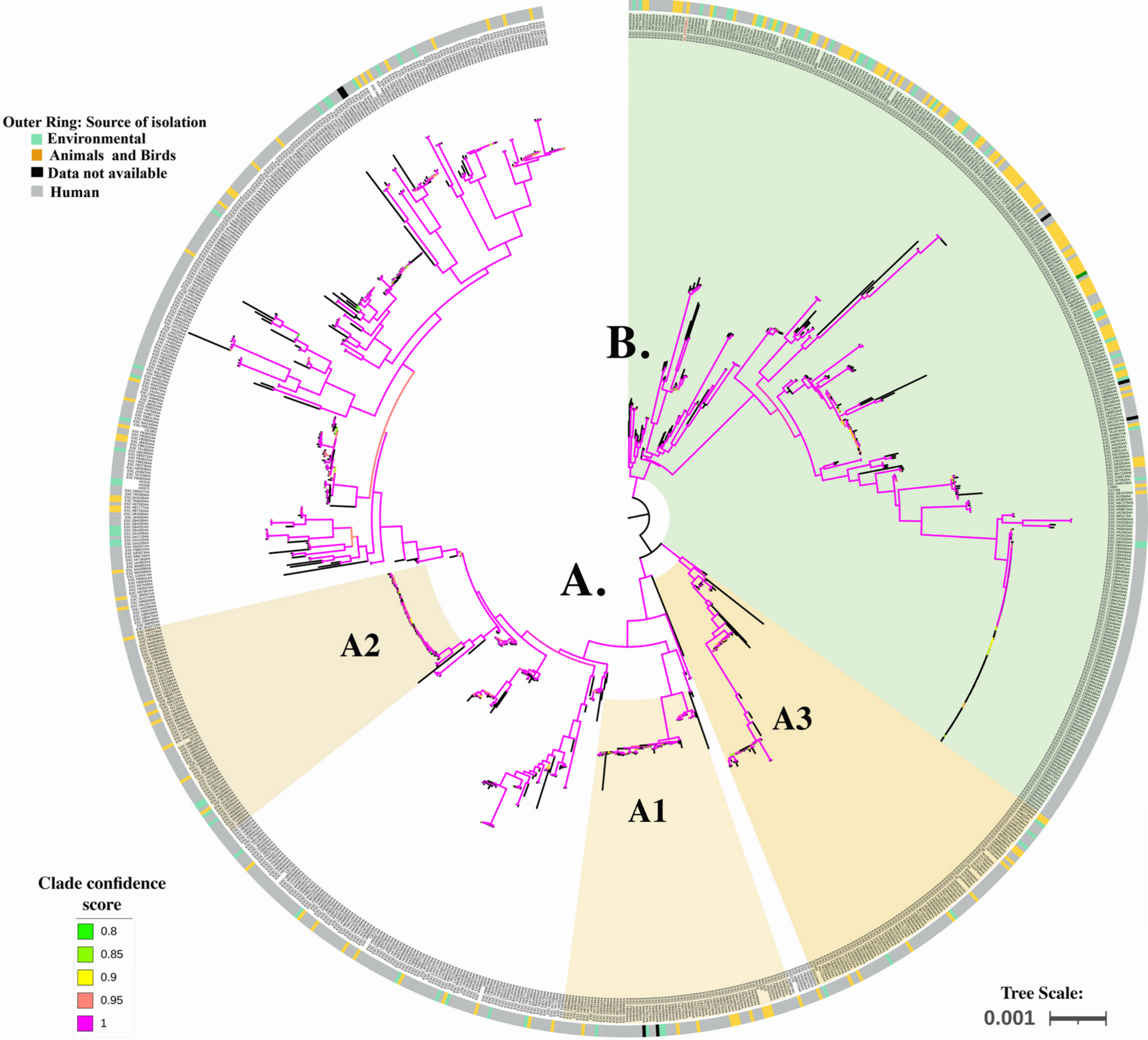

Figure S1: A midpoint rooted maximum-likelihood phylogenetic tree generated using SNIPPY as the SNP caller which supports the overall topology of the tree presented in Figure 1. The full core multi-sequence alignment, which included gaps and invariant sites, contained 5,134,443 columns (9.93% gaps, 97.41% invariant sites) with 284,857 unique columns (patterns). Relative position of genomes in clusters A1, A2, A3 of clade Aand clade B of the parSNP tree in figure 1 are also highlighted here as coloured strips.
