# Supplementary material for: Phylogenomic analysis of a global collection of Escherichia coli ST38: evidence of interspecies and environmental transmission?
Source: mSystems. 2023 Sep 7;8(5):e01236-22. doi: 10.1128/msystems.01236-22 (PMC10654095; doi:10.1128/msystems.01236-22)
Supplement: Figure S2 — Midpoint rooted maximum-likelihood phylogenetic tree representing geneology of 70 isolates in cluster A1. [file msystems.01236-22-s0002.pdf]

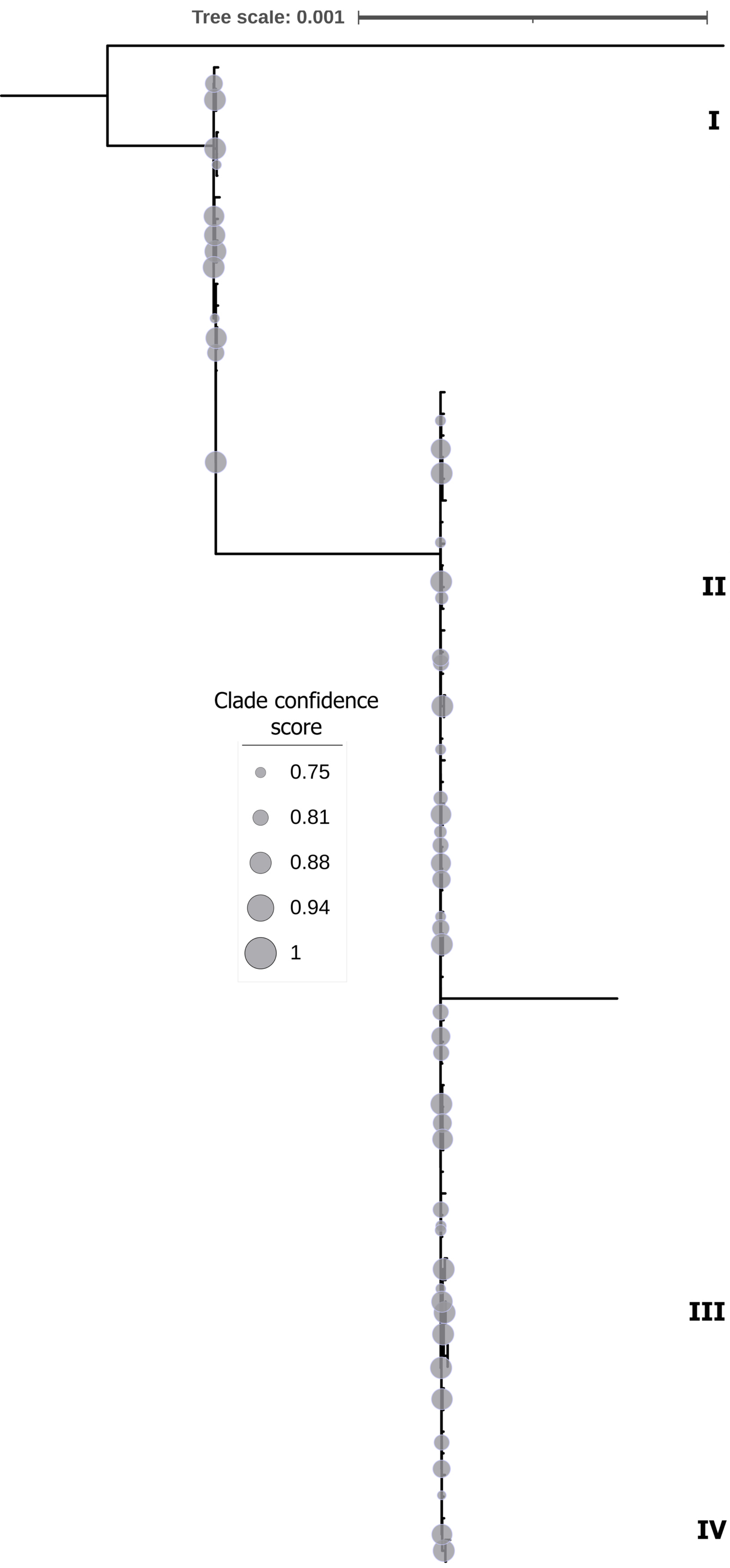

Cluster	Genomes	Integrons	Country	Source	CTXM	OXA	Plasmid Profile	SNP differences
	NZ_CP023364		Scotland	Canine			NP	0
	ESC_CB7213AA	*	Australia	Human	blaCTX-M-14	blaOXA-48	NP	5089
	ESC_RA0380AA		Sweden	Human	blaCTX-M-14	NA	NP	5065
I	ESC_PA4047AA		Sweden	Human	blaCTX-M-14	NA	NP	5069
	MVC4	*	Australia	Dog	blaCTX-M-14	NA	NP	5085
	MVC815	*	Australia	Dog	blaCTX-M-14	NA	NP	5071
	MVC82		Australia	Dog	blaCTX-M-14	NA	NP	5079
	ESC_FB7843AA	*	China	Human	blaCTX-M-14	NA	IncI2Delta	5110
	ESC_FB3375AA		China	Human	blaCTX-M-14	NA	NP	5087
	ESC_ZA4766AA	*	Kenya	Human	blaCTX-M-14	NA	NP	5083
	ESC_FB3624AA	*	Australia	Human	blaCTX-M-14	NA	NP	5074
	ESC_IA9346AA	*	Netherlands	Human	blaCTX-M-14	NA	NP	5083
	ESC_FB8159AA	*	China	Human	blaCTX-M-14	NA	NP	5090
	ESC_YA3610AA	*	N/A	Human	blaCTX-M-14	NA	NP	5077
	ESC_YA3420AA	*	N/A	Human	blaCTX-M-14	NA	NP	5078
	ESC_IA9953AA	*	Netherlands	Human	blaCTX-M-14	NA	NP	5072
	ESC_FB5741AA	*	China	Human	blaCTX-M-14	NA	pO111_1	6894
	ESC_HB7191AA		United States	Human	blaCTX-M-14	NA	NP	6887
	ESC_FB5063AA		China	Human	blaCTX-M-14	NA	NP	6881
	ESC_JA0636AA	*	Netherlands	Human	blaCTX-M-14	NA	ColMG828/IncFII	6876
	ESC_FB3455AA	*	China	Human	blaCTX-M-14	NA	ColpVC/IncFII/IncI1Gamma/IncN/IncX4/IncY	6887
	ESC_FB5048AA	*	China	Human	blaCTX-M-14/blaCTX-M-55	NA	IncFIB/IncFII/IncI1Gamma/IncN/IncX4/IncY	6906
	ESC_PA7819AA	*	United States	Human	blaCTX-M-14	NA	IncB/IncK/IncM1/IncX4	6871
	ESC_GB5995AA	*	United States	Human	blaCTX-M-14	NA	NP	6891
	ESC_WA8254AA	*	N/A	Human	blaCTX-M-14	NA	NP	6883
II	ESC_MA2356AA		Japan	Water	blaCTX-M-14	NA	IncFII/IncI2Delta/pO111_1	6886
	ESC_FB9093AA	*	Canada	Human	blaCTX-M-14	NA	NP	6881
	ESC_KB1868AA	*	Switzerland	ND	blaCTX-M-14	NA	NP	6890
	ESC_HA9590AA		Germany	Human	blaCTX-M-14	NA	NP	6873
	ESC_VA4658AA	*	Sri Lanka	Human	blaCTX-M-14	NA	pO111_1	6877
	ESC_GB6061AA	*	United States	Human	blaCTX-M-14	NA	NP	6899
	ESC_GB6062AA	*	United States	Human	blaCTX-M-14	NA	NP	6890
	ESC_IA9802AA	*	Netherlands	Human	blaCTX-M-14	NA	NP	6876
	ESC_GB9253AA		N/A	Human	blaCTX-M-14	NA	NP	6895
	ESC_NA2644AA	*	United States	Human	blaCTX-M-14	NA	IncB/O/K/IncFII	6875
	ESC_GB8596AA		N/A	Human	blaCTX-M-14	NA	NP	6890
	ESC_GB5455AA	*	N/A	Human	blaCTX-M-14	NA	NP	6874
	ESC_FB8150AA	*	China	Human	blaCTX-M-14/blaCTX-M-15	NA	IncB/O/K/IncFIB/IncFII/IncL/IncM2	6872
	ESC_FB0890AA	*	N/A	Human	blaCTX-M-14	NA	IncFIB	6868
	ESC_FB5746AA	*	China	Human	blaCTX-M-14	NA	IncFIB	6872
	ESC_CB9484AA	*	Australia	Human	blaCTX-M-14	NA	NP	6878
	ESC_GB5997AA		United States	Human	blaCTX-M-14	NA	NP	6877
	ESC_GB6032AA		United States	Human	blaCTX-M-14	NA	NP	6879
	ESC_IA9085AA	*	Netherlands	Human	blaCTX-M-14	NA	NP	6874
	ESC_FB5066AA	*	China	Human	blaCTX-M-14	NA	NP	7612
	ESC_WA7250AA	*	Cambodia	ND	blaCTX-M-14	NA	NP	6886
	ESC_IB9079AA		Australia	Human	blaCTX-M-14	NA	NP	6874
	ESC_RA0236AA	*	Sweden	Human	blaCTX-M-14	NA	NP	6869
	ESC_NA8326AA	*	Ireland	Human	blaCTX-M-14	blaOXA-48	IncL	6886
	ESC_YA3464AA	*	N/A	Human	blaCTX-M-14	NA	NP	6877
	ESC_IA8973AA	*	Netherlands	Human	blaCTX-M-14	NA	NP	6879
	ESC_JA0438AA	*	Netherlands	Human	blaCTX-M-14	NA	NP	6882
	ESC_RA7057AA	*	Netherlands	Human	blaCTX-M-14	NA	IncFII/IncX4	6875
	ESC_HA7544AA	*	Japan	Water	blaCTX-M-14	NA	NP	6918
	ESC_IA9518AA	*	Netherlands	Human	blaCTX-M-14	NA	NP	6869
	ESC_XA1174AA	*	Canada	Human	blaCTX-M-14	NA	IncFIA/IncHIA/IncHIB	6867
	ESC_QA4905AA	*	Brazil	Canine	blaCTX-M-14	NA	NP	6917
	ESC_QA4912AA	*	Brazil	Canine	blaCTX-M-14	NA	NP	6901
III	ESC_HB5878AA	*	Brazil	Human	blaCTX-M-14	NA	NP	6903
	ESC_GB0221AA	*	Brazil	Human	blaCTX-M-14	NA	NP	6904
	ESC_HB5876AA	*	Brazil	Human	blaCTX-M-14	NA	NP	6925
	ESC_GB0115AA	*	Brazil	Human	blaCTX-M-14	NA	NP	6925
	ESC_WA8474AA	*	N/A	Human	blaCTX-M-14	NA	NP	6884
	ESC_WA5787AA	*	N/A	Human	blaCTX-M-14	blaOXA-244	NP	6881
	ESC_TA7233AA	*	Canada	Human	blaCTX-M-14	NA	IncX4	6882
	ESC_UA7278AA	*	Sweden	Human	blaCTX-M-14	NA	NP	6883
	ESC_GB6057AA		United States	Human	blaCTX-M-14	NA	NP	6895
	ESC_GB3907AA		Canada	Human	blaCTX-M-14	NA	NP	6882
IV	ESC_CB2280AA	*	France	Human	blaCTX-M-14	NA	NP	6888
	ESC_TA2197AA		Australia	Avian	blaCTX-M-14	NA	NP	6968
	ESC_TA2324AA		Australia	Avian	blaCTX-M-14	NA	NP	6913

Figure S2: A midpoint rooted maximum-likelihood tree representing geneology of 70 isolates in cluster A1. Seventy eight percent of the reference genome aligned to all genomes included in the analysis and 9,680 variant sites were used to infer the genealogy of isolates. The column in the extreme right with heading 'SNP-differences' indicates the number of SNP differences with the reference genome (accession number: NZ_CP023364.1). Blue coloured strips indicate numbered transmission clusters which have been identified as clonal lineage groups in this study.
