# Supplementary material for: Phylogenomic analysis of a global collection of Escherichia coli ST38: evidence of interspecies and environmental transmission?
Source: mSystems. 2023 Sep 7;8(5):e01236-22. doi: 10.1128/msystems.01236-22 (PMC10654095; doi:10.1128/msystems.01236-22)
Supplement: Figure S4 — Midpoint rooted maximum-likelihood phylogenetic tree representing geneology of 85 isolates in cluster A3. [file msystems.01236-22-s0004.pdf]

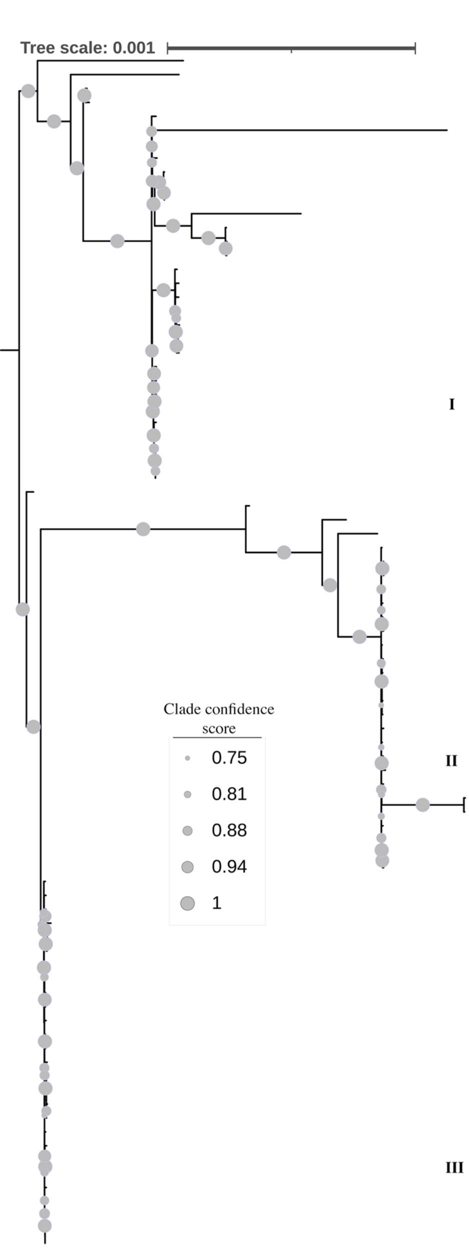

Genomes	Country	Source	Integron	CTX-M	OXA	Plasmid Replicon	SNP difference
ESC_TA7730AA	United States	Human		NA	NA	IncFIB/IncFII	5284
ESC_YA3535AA	N/A	Human		NA	blaOXA-1	ColBS512/IncFIA/IncFIB/pO111_1	4811
ESC_BA9529AA	United States	Human	*	NA	NA	IncFIA/IncFIB	3197
EC274	Australia	Human		NA	NA	ColBS512/IncFIA/IncFIB/IncFII	3231
ESC_GB6484AA	Singapore	Human		NA	NA	IncFIA/IncFIB/IncFII	2194
ESC_TA1825AA	N/A	Human		blaCTX-M-55	NA	ColBS512/IncFIA/IncFIB/IncFII/IncI1Gamma/IncP1	6024
ESC_GA8913AA	United Kingdom	Human	*	NA	NA	ColBS512/IncFIB/IncFII	2173
ESC_TA1975AA	N/A	Human		NA	NA	ColBS512/IncFIB/IncFII	2184
ESC_IA9829AA	Netherlands	Human		blaCTX-M-15	blaOXA-1	ColBS512/IncFIA/IncFIB/IncFII/IncX1	2256
ESC_YA4499AA	N/A	Human		blaCTX-M-15	NA	ColBS512/ColMG828/IncFIA/IncFIB/IncX1	2256
ESC_YA4501AA	N/A	Human		blaCTX-M-15	NA	ColBS512/ColMG828/IncFIA/IncFIB/IncFII/IncX1	2255
NZ_CP023364	Scotland	Canine				NP	0
ESC_BB5638AA	N/A	Avian		NA	NA	IncFIB/IncFII/IncX1	2124
ESC_AB7773AA	United States	Air		NA	NA	IncFIB/IncFII/IncI1Gamma	2133
ESC_HB6082AA	United States	Avian		NA	NA	Col4401/IncFIB/IncFII/IncI1Gamma	2127
ESC_FB9412AA	Canada	Human	*	blaCTX-M-14	NA	IncFIA/IncFIB/IncFII	2516
ESC_FB9417AA	Canada	Human		blaCTX-M-14	NA	pO111_1	2544
ESC_GB4259AA	N/A	Water/River		blaCTX-M-14	NA	IncFIA/IncFIB/IncFII/pO111_1	2537
ESC_GB9759AA	Canada	Human		blaCTX-M-14	NA	ColMG828/ColpVC/IncB/O/K/IncFIA/IncFIB/IncFII	2556
ESC_IA9682AA	Netherlands	Human		blaCTX-M-14	NA	IncK/IncFIA/IncFIB/IncFII	2553
ESC_TA1899AA	N/A	Human		blaCTX-M-14	NA	IncFIA/IncFIB/IncFII	2530
ESC_IB8492AA	Japan	Human	*	blaCTX-M-14/blaCTX-M-2	NA	IncFIA/IncFIB/IncFII/IncI1Gamma/IncN	2544
ESC_OA0099AA	United States	Human	*	NA	NA	ColBS512/IncB/O/K/IncFIB/IncFII	2203
ESC_CB2406AA	France	Human	*	blaCTX-M-3	NA	ColBS512/IncFIB/IncFII/IncI1Gamma	2202
EC36	Australia	Human	*	blaCTX-M-15	NA	ColBS512/ColMP18/IncFIB/IncFII/IncI1Gamma	2199
ESC_XA7257AA	Vietnam	Human	*	NA	NA	ColBS512/IncFIB/IncFII/IncI1Gamma	2201
ESC_TA7554AA	United States	Human		NA	NA	ColBS512/IncFIB/IncFII/IncX4	2192
ESC_TA7553AA	United States	Human		NA	NA	ColBS512/IncFIB/IncFII	2188
ESC_TA7608AA	United States	Human	*	NA	NA	ColBS512/IncM1/IncX4	2186
ESC_TA7745AA	United States	Human	*	NA	NA	ColBS512/IncM1/IncX4	2188
ESC_TA7746AA	United States	Human		NA	NA	ColBS512/IncM1/IncX4	2187
ESC_SA2844AA	United Kingdom	Human		NA	NA	ColBS512/IncFIB/IncFII	4501
ESC_CB9398AA	Australia	Human	*	blaCTX-M-55	NA	IncFIA/IncFIB/IncFII	6588
ESC_FB3582AA	Australia	Human		NA	blaOXA-1	ColBS512/IncFIA/IncFIB	6427
ESC_TA2235AA	Australia	Avian		blaCTX-M-15	blaOXA-1	ColMG828/IncFIA/IncFIB/IncFIB/IncFII	5969
ESC_FA6828AA	Mali	Human	*	NA	blaOXA-1	IncFIA/IncFIB/IncFII	6547
ESC_RA8250AA	United States	Human		NA	blaOXA-1	IncFIA/IncFIB/IncX4	6574
ESC_RA8222AA	United States	Human	*	NA	blaOXA-1	IncFIA/IncFIB/IncX4	6575
ESC_PA1731AA	Vietnam	Human	*	blaCTX-M-27	blaOXA-1	ColMG828/IncFIA/IncFIB/IncFII/IncY	6551
ESC_JB1176AA	United States	Human	*	NA	blaOXA-1	IncFIA/IncFIB	6560
ESC_FB3602AA	Australia	Human		NA	NA	IncFIA/IncFIB	6562
ESC_FB9521AA	New Zealand	Human		NA	blaOXA-1	IncFIA/IncFIB/IncFII	6567
ESC_TA7635AA	United States	Human	*	NA	NA	IncFIA	6550
ESC_GB9358AA	N/A	Human		blaCTX-M-14	NA	IncFIA/IncFIB/IncFII/IncI1Gamma/IncX4	6563
ESC_XA1124AA	Canada	Human		blaCTX-M-14	NA	NP	6557
ESC_CB2330AA	France	Human	*	blaCTX-M-14	NA	IncFIB	6576
ESC_GB4180AA	Canada	Human	*	blaCTX-M-14	blaOXA-1	ColBS512/IncFIA/IncFIB/IncFII	6560
ESC_WA8838AA	N/A	Human	*	blaCTX-M-14	blaOXA-1	IncFIA/IncFIB	6559
ESC_XA1064AA	Canada	Human		blaCTX-M-55	NA	Col8282/IncI1Gamma/IncX1	6568
ESC_FB3094AA	Canada	Human	*	blaCTX-M-15	NA	ColBS512/IncFIA/IncFIB/IncFII/IncN	6548
HOS59	Australia	Human	*	blaCTX-M-14	NA	IncB/O/K/IncFIA/IncFIB/IncFII	6548
ESC_JB8356AA	China	Human	*	blaCTX-M-14	NA	ColKPHS6/IncFIA/IncFIB/IncFII/IncL/IncN	6583
ESC_GB0205AA	New Zealand	Human		blaCTX-M-15	NA	IncFIA/IncFIB/IncFII/IncI1Gamma	6567
ESC_UA3515AA	Switzerland	Human	*	NA	blaOXA-1	ColBS512/IncFIA/IncFIB/IncFII	7766
ESC_CB7222AA	Australia	Human	*	NA	blaOXA-181	Col156/IncFIA/IncFIB/IncX3/IncX4	7756
ESC_GB6565AA	Malaysia	Human	*	blaCTX-M-27	NA	ColMG828/IncFIA/IncFIB/IncFII/IncI1Gamma/pO111_1	6560
ESC_XA8562AA	Singapore	Human	*	NA	blaOXA-1	ColBS512/IncB/O/K/IncFIA/IncFIB	6558
ESC_PA1115AA	Australia	Human		NA	NA	ColBS512/IncFIA/IncFIB	6561
ESC_RA5020AA	Germany	Human	*	NA	NA	ColBS512/IncFIA/IncFIB	6568
ESC_RA7051AA	Germany	Human		blaCTX-M-15	NA	ColBS512/IncB/O/K/IncFII/IncY	4584
ESC_YA3435AA	N/A	Human		NA	NA	ColpVC/IncFIA/IncFIB/IncFII	4596
ESC_OA0341AA	Canada	Human	*	NA	blaOXA-48	IncFIA/IncFIB/IncFII/IncL/IncX4	4594
ESC_GB7014AA	United States	Human	*	blaCTX-M-15	NA	ColBS512/IncFIA/IncFIB/IncFII/IncI1Gamma/IncY	4673
GH_47	United Kingdom	Water	*	blaCTX-M-15	NA	ColMG828/IncFIA/IncFIB/IncFII/IncFII/IncX4	4775
ESC_OA6281AA	United States	Human	*	blaCTX-M-15	NA	IncFIA/IncFIB/IncFII	4612
ESC_IB7426AA	Canada	Human		NA	NA	IncFII	4601
ESC_FB3555AA	Australia	Human		blaCTX-M-15	NA	ColMG828/IncFIA/IncFIB/IncFII/IncI1Gamma	4608
ESC_PA1702AA	Vietnam	Human	*	blaCTX-M-27	NA	ColMG828/IncFIA/IncFIB/IncFII	4589
ESC_PA1715AA	Vietnam	Human	*	blaCTX-M-27	NA	ColBS512/ColMG828/IncFIA/IncFIB/IncFII	4588
ESC_FA7252AA	United States	Human	*	NA	NA	IncFIA/IncFIB/IncFII	4595
ESC_JA0759AA	Netherlands	Human		blaCTX-M-27	NA	IncFIA/IncFIB/IncFII/IncM1	4589
ESC_JA0698AA	Netherlands	Human		blaCTX-M-27	NA	IncFIA/IncFIB/IncFII/IncM1	4589
ESC_FB9453AA	New Zealand	Human		blaCTX-M-27	NA	IncFIA/IncFIB/IncFII	4611
ESC_GB5982AA	United States	Human	*	blaCTX-M-27	NA	ColMG828/IncFIA/IncFIB/IncFIB/IncFII/IncY	4601
ESC_GB5993AA	United States	Human	*	blaCTX-M-27	NA	ColMG828/IncFIA/IncFIB/IncFIB/IncFII/IncY	4601
ESC_GB6064AA	United States	Human	*	blaCTX-M-27	NA	ColMG828/IncFIA/IncFIB/IncFIB/IncFII/IncY	4616
ESC_GB6045AA	United States	Human	*	blaCTX-M-27	NA	ColMG828/IncFIA/IncFIB/IncFIB/IncFII/IncY	4603
ESC_XA6569AA	Singapore	Human	*	blaCTX-M-15	blaOXA-1	ColBS512/ColMG828/Col156/IncFIA/IncFIB/IncFII/IncI1Gamma/IncX4	4589
ESC_IB9015AA	Australia	Human	*	blaCTX-M-27	NA	IncFIA/IncFIB/IncFII	4605
ESC_MA2322AA	Japan	Water	*	blaCTX-M-27	NA	IncFIA/IncFIB/IncFII	4600
ESC_YA1551AA	Switzerland	Human	*	NA	NA	IncFIA/IncFIB/IncFII	4599
ESC_FB9668AA	New Zealand	Human	*	blaCTX-M-27	NA	IncB/IncK/IncFIA/IncFIB/IncFII/IncX4	4599
ESC_GB5486AA	N/A	Human	*	blaCTX-M-27	NA	IncFIA/IncFIB/IncFII	4597
ESC_PA1722AA	Vietnam	Human	*	blaCTX-M-15	NA	IncFIA/IncFIB/IncFII/IncI1Gamma/IncX4	4586
ESC_PA1668AA	Vietnam	Human	*	blaCTX-M-27	NA	IncFIA/IncFIB/IncFII	4582
ESC_PA1667AA	Vietnam	Human	*	blaCTX-M-27	NA	IncFIA/IncFIB/IncFII	4581

Figure S4: A midpoint rooted maximum-likelihood tree representing geneology of 85 isolates in cluster A3. Eighty percent of the reference genome aligned to all genomes included in the analysis and 18,285 variant sites were used to infer the genealogy of isolates. The column in the extreme right with heading 'SNP-differences' indicates the number of SNP differences with the reference genome (accession number: NZ_CP023364.1). Blue coloured strips indicate numbered transmission clusters which have been identified as clonal lineage groups in this study.
