# Supplementary figures and images for: Phylogenomic analysis of a global collection of Escherichia coli ST38: evidence of interspecies and environmental transmission?
Source: mSystems. 2023 Sep 7;8(5):e01236-22. doi: 10.1128/msystems.01236-22 (PMC10654095; doi:10.1128/msystems.01236-22)

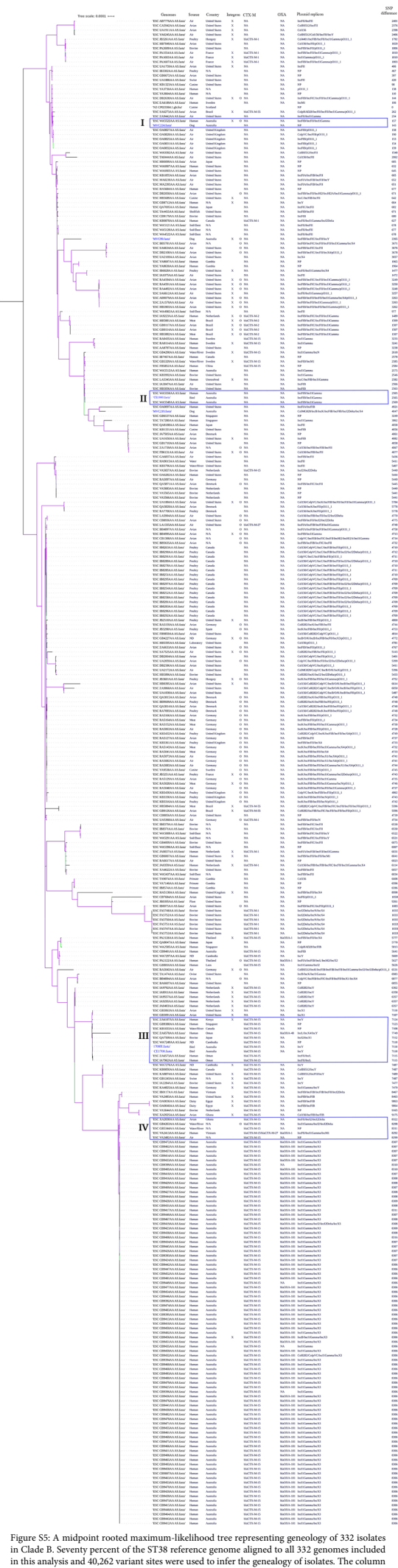

Supplement: Figure S5 — Midpoint rooted maximum-likelihood phylogenetic tree representing geneology of 332 isolates in clade B. [file msystems.01236-22-s0005.pdf]
