# Supplementary material for: Phylogenomic analysis of a global collection of Escherichia coli ST38: evidence of interspecies and environmental transmission?
Source: mSystems. 2023 Sep 7;8(5):e01236-22. doi: 10.1128/msystems.01236-22 (PMC10654095; doi:10.1128/msystems.01236-22)
Supplement: Figure S3 — Midpoint rooted maximum-likelihood phylogenetic tree representing geneology of 67 isolates in cluster A2. [file msystems.01236-22-s0003.pdf]

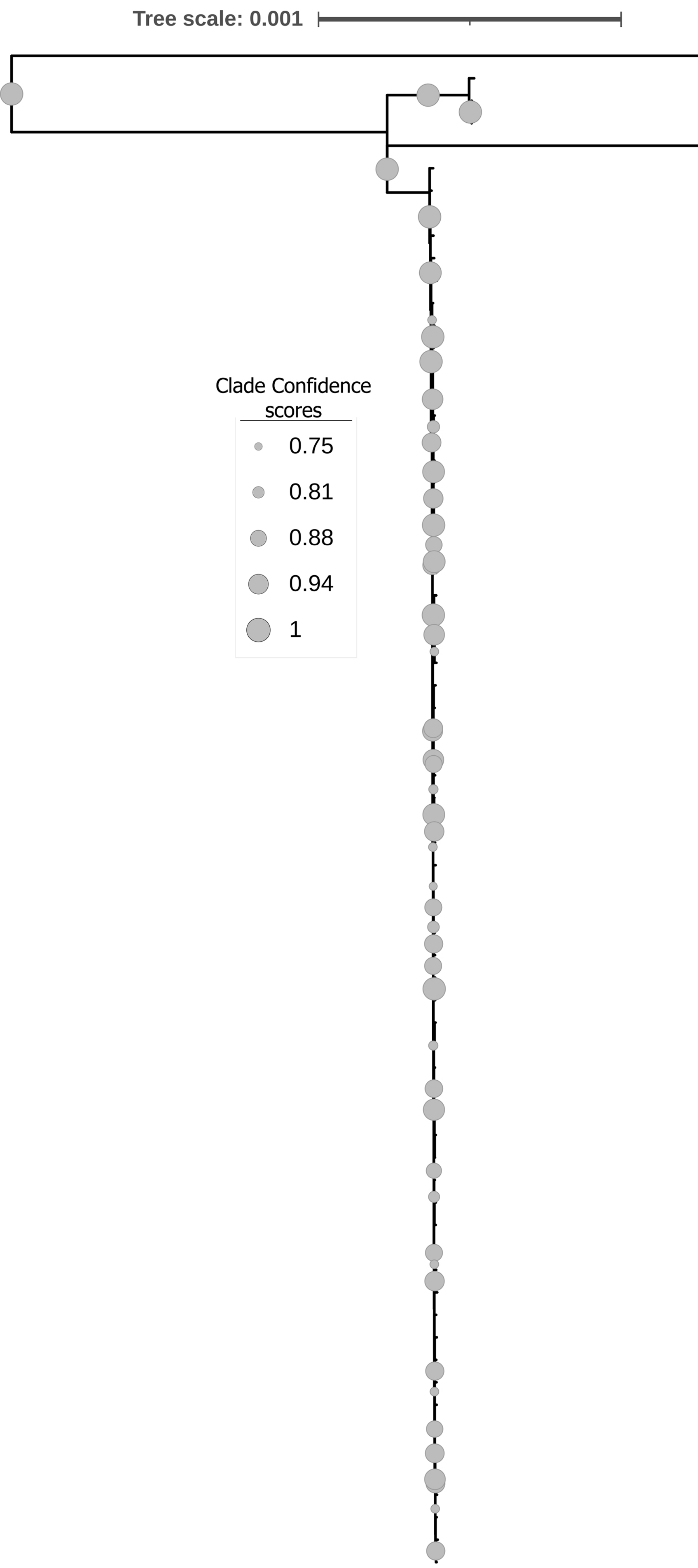

Cluster	Genomes	Country	Source	Integron	CTX-M	OXA	Plasmid Profile	SNP differences
	NZ_CP023364	Scotland	Canine				NP	0
	ESC_YA3500AA	N/A	Human		NA	NA	IncFIA/IncFIB/IncFIC/IncFII	7224
	ESC_WA8895AA	N/A	Human		NA	NA	IncFIA/IncFIB/IncFIC/IncFII	7196
	ESC_WA8845AA	N/A	Human		NA	NA	IncFIA/IncFIB/IncFIC/IncFII/IncFII	7195
	ESC_GB8666AA	N/A	Human	*	blaCTX-M-14	NA	ColBS512/ColMG828/Col156/IncFIA/IncFIB/IncFII	7316
	ESC_GB3692AA	N/A	Human		NA	NA	IncFIA/IncFIB/IncFIB/IncFIC/IncFII	7059
	ESC_XA1164AA	Canada	Human		NA	NA	IncFIA/IncFIB/IncFIC/IncFII	7040
	ESC_GB6588AA	Singapore	Human		NA	NA	IncFIA/IncFIB/IncFIC/IncFII	7067
	ESC_CB2284AA	France	Human		blaCTX-M-15	NA	IncFIA/IncFIB/IncFIB/IncFIC/IncFII	7058
	ESC_GA1823AA	United States	Water		NA	NA	IncFIA/IncFIB/IncFIC/IncFII	7074
	ESC_IB7623AA	Canada	Human		NA	NA	IncFIA/IncFIB/IncFIC/IncFII	7110
	ESC_GB6598AA	Singapore	Human	*	blaCTX-M-27	NA	IncFIB/IncFII	7055
	ESC_PA1664AA	Vietnam	Human	*	blaCTX-M-27	NA	IncFIB/IncFII	7070
	ESC_PA1663AA	Vietnam	Human	*	blaCTX-M-27	NA	IncFIB/IncFII	7061
	ESC_GB5514AA	N/A	Human	*	blaCTX-M-27	NA	IncFIB/IncFIB/IncFII	7078
	ESC_CB2341AA	France	Human	*	blaCTX-M-27	NA	IncFIB/IncFII	7072
	ESC_RA0458AA	Sweden	Human		NA	NA	IncFIB	7067
	ESC_XA7353AA	Norway	Human		blaCTX-M-27	NA	IncFIB/IncFII	7065
	ESC_JA0320AA	Netherlands	Human	*	blaCTX-M-27	NA	IncFIB/IncFII	7065
	ESC_IA9532AA	Netherlands	Human	*	blaCTX-M-27	NA	IncFIB/IncFII	7059
	ESC_WA8826AA	N/A	Human	*	blaCTX-M-27	NA	IncFIB/IncFIB/IncFII	7074
	ESC_WA8932AA	N/A	Human	*	blaCTX-M-27	NA	IncFIB/IncFII	7069
	ESC_WA8938AA	N/A	Human		blaCTX-M-27	NA	IncFIB/IncFII	7064
	ESC_WA9001AA	Ecuador	Human		blaCTX-M-27	NA	IncFIB/IncFII	7074
	ESC_FB6937AA	China	Human	*	blaCTX-M-27	NA	IncB/O/K/IncFII/IncY	7087
	ESC_FB7844AA	China	Human	*	blaCTX-M-27	NA	IncFII	7077
	ESC_FB6938AA	China	Human	*	blaCTX-M-27	NA	IncB/O/K/IncFII	7089
	ESC_XA7274AA	Vietnam	Human	*	blaCTX-M-27	NA	IncFII	7089
	ESC_GB9233AA	N/A	Human		blaCTX-M-27	NA	IncFIB/IncFII	7067
I	ESC_JB8396AA	Australia	Avian		blaCTX-M-27	NA	IncFIB/IncFII	7066
I	ESC_FB7958AA	China	Human	*	blaCTX-M-27	NA	IncFIB/IncFII	7086
	ESC_GB9369AA	Singapore	Human	*	blaCTX-M-27	NA	IncFIB/IncFII/IncY	7080
	ESC_FB5840AA	China	Human	*	blaCTX-M-27	NA	IncFIB/IncFII	7072
	ESC_TA7197AA	Singapore	Human	*	blaCTX-M-27	NA	IncFIB/IncFIB	7062
	ESC_TA7223AA	Singapore	Human	*	blaCTX-M-27	NA	IncFIB	7061
	ESC_GB6102AA	Singapore	Human	*	blaCTX-M-27	NA	IncFIB/IncFII	7068
	ESC_FB1295AA	China	Human		blaCTX-M-27	NA	IncFIB/IncFII/IncFII	7079
II	ESC_FB9365AA	Canada	Human		blaCTX-M-27	NA	ColBS512/IncFIB/IncFII	7063
II	ESC_UA7319AA	Sweden	Human	*	blaCTX-M-27	NA	IncFIB/IncFII	7064
II	EC231	Australia	Human	*	blaCTX-M-27	NA	IncFIB	7069
	ESC_GB8716AA	N/A	Human		blaCTX-M-27	NA	IncFIB/IncFII/IncFII	7066
	ESC_GB0138AA	Brazil	Human		blaCTX-M-27	NA	IncFIB/IncFII	7070
	ESC_HB5877AA	Brazil	Human		blaCTX-M-27	NA	IncFIB/IncFII	7074
	ESC_CB2305AA	France	Human	*	blaCTX-M-27	NA	IncFIB/IncFII	7066
	ESC_CB2323AA	France	Human	*	blaCTX-M-27	NA	IncFIB/IncFII	7075
	ESC_PA2118AA	Thailand	Human	*	blaCTX-M-27	NA	IncFIB/IncFIB/IncFII	7071
	ESC_CB2257AA	France	Human		blaCTX-M-27	NA	IncFIB	7087
	ESC_CB2318AA	France	Human		blaCTX-M-27	NA	IncFIB/IncFII/IncI1Gamma	7069
	ESC_CB2336AA	France	Human		blaCTX-M-27	NA	IncFIB/IncFIB	7076
	ESC_CB2258AA	France	Human		blaCTX-M-27	NA	IncFIB/IncI1Gamma	7069
	ESC_IB7700AA	Canada	Human		blaCTX-M-27	NA	IncFIB/IncFII	7065
	ESC_FB5848AA	China	Human	*	blaCTX-M-27	NA	IncFIB/IncFII/IncFII	7076
	ESC_FB9676AA	New Zealand	Human	*	blaCTX-M-27	NA	IncFIB/IncFII	7074
	ESC_JB9713AA	China	Human	*	blaCTX-M-27/blaCTX-M-55	NA	IncFIB/IncFII/IncI1Gamma	7072
III	ESC_RA6707AA	United States	Water	*	blaCTX-M-27	NA	IncFIB/IncFII	7074
III	ESC_GB9439AA	Singapore	Human		blaCTX-M-27	NA	IncFIB/IncFII	7086
	ESC_FB3728AA	Australia	Human		blaCTX-M-27	NA	IncFIB/IncFII	7076
	ESC_RA0217AA	Sweden	Human		blaCTX-M-27	NA	IncFIB	7084
	ESC_FB9546AA	New Zealand	Human		blaCTX-M-27	NA	IncB/O/K/IncFIB/IncFII	7076
	ESC_JB3117AA	France	Human		blaCTX-M-27	NA	IncFIB/IncFII/IncI1Gamma	7078
	ESC_IB9063AA	Australia	Human	*	blaCTX-M-27	NA	IncFIB/IncFII	7081
	ESC_GB6533AA	Thailand	Human	*	blaCTX-M-27	NA	ColBS512/IncFIB/IncFII	7076
	ESC_XA7366AA	Norway	Human	*	blaCTX-M-27	NA	IncFIB/IncFII	7074
	ESC_CB2411AA	France	Human	*	blaCTX-M-27	NA	IncFIB/IncFII	7061
	ESC_GB8593AA	N/A	Human	*	blaCTX-M-27	NA	IncFIB/IncFII	7076
	ESC_CB9397AA	Australia	Human		blaCTX-M-27	NA	IncFIB/IncFII	7079
	ESC_BB4486AA	Germany	Human	*	blaCTX-M-27	blaOXA-244	IncFIB/IncFII	7072
	ESC_GB5470AA	N/A	Human	*	blaCTX-M-27	blaOXA-244	IncFIB/IncFII	7066

Figure S3: A midpoint rooted maximum-likelihood tree representing geneology of 67 isolates in cluster A2. Eighty three percentage of the reference genome aligned to all genomes included in the analysis and 10,910 variant sites were used to infer genealogy of isolates. The column in the extreme right with heading 'SNP-differences' indicates the number of SNP differences with the reference genome (accession number: NZ_CP023364.1). Blue coloured strips indicate numbered transmission clusters which have been identified as clonal lineage groups in this study.
